# Anaerobic gut fungi are an untapped reservoir of natural products

**DOI:** 10.1073/pnas.2019855118

**Published:** 2021-04-27

**Authors:** Candice L. Swift, Katherine B. Louie, Benjamin P. Bowen, Heather M. Olson, Samuel O. Purvine, Asaf Salamov, Stephen J. Mondo, Kevin V. Solomon, Aaron T. Wright, Trent R. Northen, Igor V. Grigoriev, Nancy P. Keller, Michelle A. O’Malley

**Affiliations:** ^a^Department of Chemical Engineering, University of California, Santa Barbara, CA 93106;; ^b^Department of Energy Joint Genome Institute, Lawrence Berkeley National Laboratory, Berkeley, CA 94720;; ^c^Earth and Biological Sciences Division, Pacific Northwest National Laboratory, Richland, WA 99352;; ^d^Environmental Molecular Sciences Laboratory, Pacific Northwest National Laboratory, Richland, WA 99352;; ^e^Department of Agricultural Biology, Colorado State University, Fort Collins, CO, 80523;; ^f^Department of Agricultural and Biological Engineering, Purdue University, West Lafayette, IN 47907;; ^g^Environmental Genomics and Systems Biology Division, Lawrence Berkeley National Laboratory, Berkeley, CA 94720;; ^h^Joint BioEnergy Institute, Emeryville, CA 94608;; ^i^Department of Plant and Microbial Biology, University of California, Berkeley, CA 94720;; ^j^Department of Medical Microbiology and Immunology and Bacteriology, University of Wisconsin–Madison, Madison, WI 53706

**Keywords:** natural products, secondary metabolism, anaerobes, fungi, transcriptomics

## Abstract

Anaerobic fungi (class Neocallimastigomycetes) thrive as low-abundance members of the herbivore digestive tract. The genomes of anaerobic gut fungi are poorly characterized and have not been extensively mined for the biosynthetic enzymes of natural products such as antibiotics. Here, we investigate the potential of anaerobic gut fungi to synthesize natural products that could regulate membership within the gut microbiome. Complementary 'omics' approaches were combined to catalog the natural products of anaerobic gut fungi from four different representative species: *Anaeromyces robustus *(*A*. *robustus*), *Caecomyces churrovis *(*C*. *churrovis*), *Neocallimastix californiae *(*N*. *californiae*), and *Piromyces finnis* (*P*. *finnis*). In total, 146 genes were identified that encode biosynthetic enzymes for diverse types of natural products, including nonribosomal peptide synthetases and polyketide synthases. In addition, *N. californiae* and *C. churrovis* genomes encoded seven putative bacteriocins, a class of antimicrobial peptides typically produced by bacteria. During standard laboratory growth on plant biomass or soluble substrates, 26% of total core biosynthetic genes in all four strains were transcribed. Across all four fungal strains, 30% of total biosynthetic gene products were detected via proteomics when grown on cellobiose. Liquid chromatography-tandem mass spectrometry (LC-MS/MS) characterization of fungal supernatants detected 72 likely natural products from *A. robustus* alone. A compound produced by all four strains of anaerobic fungi was putatively identified as the polyketide-related styrylpyrone baumin. Molecular networking quantified similarities between tandem mass spectrometry (MS/MS) spectra among these fungi, enabling three groups of natural products to be identified that are unique to anaerobic fungi. Overall, these results support the finding that anaerobic gut fungi synthesize natural products, which could be harnessed as a source of antimicrobials, therapeutics, and other bioactive compounds.

## Significance

Anaerobic gut fungi are important members of the gut microbiome of herbivores, yet they exist in small numbers relative to bacteria. Here, we show that these early-branching fungi produce a wealth of secondary metabolites (natural products) that may act to regulate the gut microbiome. We use an integrated 'omics'-based approach to classify the biosynthetic genes predicted from fungal genomes, determine transcriptionally active genes, and verify the presence of their enzymatic products. Our analysis reveals that anaerobic gut fungi are an untapped reservoir of bioactive compounds that could be harnessed for biotechnology.

Secondary metabolites, or natural products, have inspired many medicinal drugs, including antibiotics, antitumor agents, and immunosuppressants (1). In addition to pharmaceuticals, natural products have also found use as valuable bio-based products such as drop-in biofuels and renewable polymers (2, 3). Across microbial diversity, fungi are especially prolific secondary metabolite producers, with a single strain such as *Aspergillus nidulans* FGSC A4 producing 15 compounds characterized in the Minimum Information about a Biosynthetic Gene cluster database (4) or *Aspergillus fumigatus* with over 18 characterized metabolites (5). Despite the wealth of valuable polyketides and other secondary metabolites already derived from fungi, the capacity for discovery is far from realization. Certain fungal genera, such as *Aspergillus*, are disproportionately studied, although they represent only a fraction of sequenced fungal genomes (6). Rediscovery of natural products like antibiotics has proved problematic, requiring innovative approaches to silence known antibiotic-producing genes (7), or alternatively, investigation of rarely explored microbiomes, such as the rumen, for sequence-divergent biosynthetic genes. Examples of antibiotics discovered from unusual environments include lugdunin, which was discovered from a commensal bacteria of the human microbiome (8), as well as teixobactin, which was discovered from a screen of previously uncultured bacteria (9). Both lugdunin and teixobactin were active against *Staphylococcus aureus,* and teixobactin was active without detectable resistance.

Anaerobic gut fungi (class Neocallimastigomycetes) are understudied organisms that thrive as members of a consortium of archaea, bacteria, and protozoa in the digestive tracts of large herbivores (10–12). In these habitats, fungi are vastly outnumbered by prokaryotic microorganisms by several orders of magnitude (10–12). For instance, rumen bacteria are estimated at 10^10^ cells per gram rumen contents (10–12) whereas fungi are estimated at 10^6^ per gram (10–12). These fungi are of recent biotechnological interest due to their array of biomass-degrading enzymes, but through genome and transcriptome sequencing it has become evident that they also have a range of biosynthetic enzymes for natural products (13–15). We hypothesize that anaerobic gut fungi synthesize natural products to compete with other microbes for survival in their native environment. Natural products are known to serve a variety of functions to their producers in other environments, including oxidative stress tolerance (16), fungal development (17), and antibiosis (18). Similarly, the natural products of anaerobic gut fungi may serve directly (by antibiosis) or indirectly (by conferring environmental stress tolerance) to allow the fungi to persist despite being outnumbered by other members of the rumen community.

Here, we take an integrated approach combining genomics, transcriptomics, proteomics, and metabolomics to develop a pipeline to identify and characterize natural products from anaerobic gut fungi. By using antibiotics and Secondary Metabolites Analysis Shell (antiSMASH) (19), we classify the types of biosynthetic enzymes present in the fungal genomes of representative Neocallimastigomycetes and quantify the homology between strains as well as to other organisms. Transcriptomics and proteomics are used to validate and refine these predictions. Finally, we demonstrate by metabolomics and molecular networking that anaerobic gut fungi produce a polyketide-related compound baumin, as well as at least three groups of natural products.

## Results and Discussion

### The Genomes of Anaerobic Gut Fungi Encode Diverse Biosynthetic Enzymes for Natural Products and Antimicrobial Peptides.

**Fig. 1.**
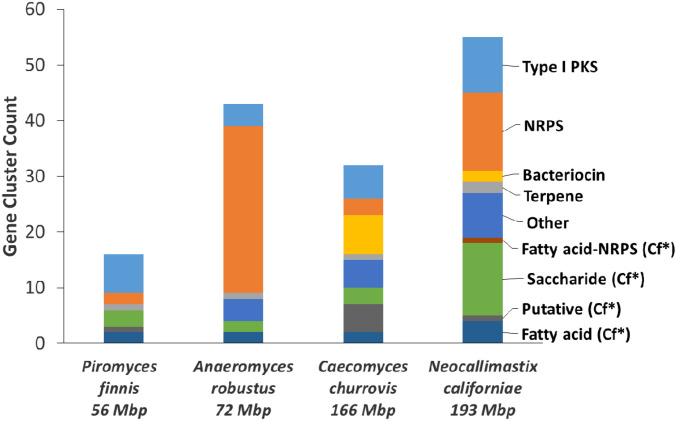
Anaerobic fungal genomes reveal putative natural products of many different types. The genomes of anaerobic fungi (13) were mined for their biosynthetic gene clusters and cluster types by antiSMASH 3.0 (19) using profile Hidden Markov Models with the ClusterFinder option (20). *Cf = gene clusters identified by ClusterFinder. The ClusterFinder algorithm extends the secondary metabolite search to include biosynthetic gene clusters of unknown types based on the occurrence of common protein family domains inside and outside of the cluster (20).

Previously, we isolated and sequenced the genomes of four species of anaerobic gut fungi from the early-branching fungal class Neocallimastigomycetes (14). Using antiSMASH version 3.0 (19) to mine the genomes of *Anaeromyces robustus*, *Caecomyces churrovis*, *Neocallimastix californiae*, and *Piromyces finnis*, we uncovered 146 genes encoding enzymes responsible for the synthesis of various classes of secondary metabolites (Fig. 1). These enzymes include canonical classes such as polyketide synthases (PKSs) and nonribosomal peptide synthetases (NRPSs), as well as putative classes based on the ClusterFinder (20) algorithm. The number of predicted core biosynthetic genes, or backbone genes, in all four strains as a proportion of total genes is commensurate with the prolific secondary metabolite-producing aspergilli (*SI Appendix*, Table S1), which contain roughly 50 to 70 backbone genes per strain. Neocallimastigomycetes surpass other members of Chytridiomycota by an order of magnitude in the number of backbone genes per strain (*SI Appendix*, Table S1).

Surprisingly, antiSMASH identified nine bacteriocins, or antimicrobial peptides (AMPs) typically produced by bacteria (21), in the genomes of anaerobic fungi: two predicted peptides were found in *N. californiae* and four unique peptide sequences were found in *C. churrovis*. We validated the antiSMASH predictions against other tools specifically designed to identify AMPs from sequence data (*SI Appendix*, Supplementary Text). Namely, we queried the six unique bacteriocin amino acid sequences using Antimicrobial Peptide Calculator and Predictor (22) and CAMPSign (23). Although none of the sequences belonged to the 45 AMP families in CAMPSign, subsequent BLAST to the AMP Databases indicated that all sequences shared at least 31.6% identity to putative bacteriocins or lactococcin 972 (24) (*SI Appendix*, Dataset S1). The bacteriocins located on *C. churrovis* scaffolds 90 and 616 and *N. californiae* scaffold 388 were transcribed but not detected in the proteome. Taken together, these results indicate that both *C. churrovis* and *N. californiae* genomes encode potential AMPs in addition to an arsenal of PKSs and NRPSs.

To further affirm the biosynthetic genes of anaerobic gut fungi, we compared the antiSMASH predictions to the secondary metabolite genes predicted by the Secondary Metabolite Unknown Regions Finder (SMURF) algorithm (25) in the Joint Genome Institute (JGI) MycoCosm portal (26) (*SI Appendix*, Dataset S2). For all strains except *A. robustus*, antiSMASH, with the ClusterFinder algorithm enabled, predicted more biosynthetic genes because it detected a wider array of natural product classes than SMURF, including bacteriocins and putative classes such as fatty acid and saccharide derivatives. For *A. robustus*, SMURF predicted an additional five PKS-like biosynthetic genes. Despite these differences (discussed in greater detail the *SI Appendix*, Supplementary Text), the majority of the regions on each scaffold predicted by antiSMASH or SMURF to harbor biosynthetic genes were the same. A total of 90% of the backbone genes predicted by SMURF in each fungal strain were located on the same or an overlapping scaffold region where antiSMASH also identified biosynthetic genes.

### The Biosynthetic Genes of Anaerobic Fungi Are Isolated or Cluster with Nonconventional Genes.

The biosynthetic enzymes of fungal secondary metabolism are typically, but not always, encoded by genes locally clustered on the chromosome with other genes in the biological pathway, such as genes that encode tailoring enzymes, transporters, self-resistance genes, and transcription factors (27, 28). AntiSMASH predicted cluster accessory genes based on GlimmerHMM (29) and up to 20 kbp intergenic distance for the outermost gene (30). Based on RNA-seq data, antiSMASH was a poor predictor of the accessory genes. In order to delineate the accessory genes of each cluster, we relied on a variety of gene prediction models, including GeneMark (31, 32) and fgenesh (33), and only included genes that were validated via RNA sequencing (*Materials and* *Methods*). The curated gene clusters are presented in the Secondary Metabolite Clusters feature of the MycoCosm portal (26) as well as in *SI Appendix*, Dataset S3.

Approximately 60% of the backbone genes with RNA-seq support are located in clusters of two or more genes, and 40% of the backbone genes are isolated (neighbored by genes greater than 10 kbp apart or by genes with poor RNA-seq coverage). For the backbone genes of anaerobic fungi that are located in clusters, some of the neighboring genes are not typically found in either bacterial or other fungal biosynthetic gene clusters. Many of the neighboring genes encode hypothetical proteins or lack any homology-based annotations. However, in some cases, the neighboring genes include solute transporters and enzymes responsible for posttranslational modifications (e.g., phosphorylation and palmitoylation), which are more commonly observed in biosynthetic gene clusters. Notably, only the PKS-like gene cluster of *P. finnis* located on scaffold 39 (core gene MycoCosm Protein Id 358210) includes a putative transcription factor (414496). However, the *A. robustus* PKS located on scaffold 258 and *N. californiae* PKS-like gene cluster on scaffold 59 both include proteins with ankyrin repeats (*A. robustus* 270780 and *N. californiae* 668532) that may be bANK family transcription factors found in several other fungi (34–36). The predicted gut fungal proteins do not match the motif of basic amino acids found in other bANK proteins (34, 35, 37), but this motif is not required for transcription factor activity (37).

Nonconventional neighboring genes that are present in more than one gene cluster include C-type lectins (*N. californiae* Protein Ids 502167 and 674020 and *P. finnis* 349079), peptidases (*C. churrovis* 519541 and *P. finnis* 241287), and calmodulin-related proteins of the EF-hand superfamily (*A. robustus* 27040 and *C. churrovis* 200925). Although the functions of these genes are unknown, it is possible that they may be self-resistance genes. Self-resistance genes have been observed in both bacterial and fungal biosynthetic gene clusters (38, 39). Another candidate self-resistance gene is *C. churrovis* 17006, encoding ribosomal protein L19e (specific to eukaryotes and archaea), which suggests that the backbone enzyme, encoded by Protein Id 17094, may synthesize a compound with activity against another eukaryote. It has been suggested, but not proven, that variant copies of the ribosomal L11 protein may be self-resistance genes for *Bacillus cereus* American Type Culture Collection 14579, which is a producer of thiocillin (40). The function of these nonconventional neighboring genes in the gene clusters of anaerobic fungi and whether they have a role in gut fungal secondary metabolism remains to be determined.

### Biosynthetic Gene Sequences Support Horizontal Gene Transfer from Other Rumen Microbes as a Mechanism of Acquisition.

**Fig. 2.**
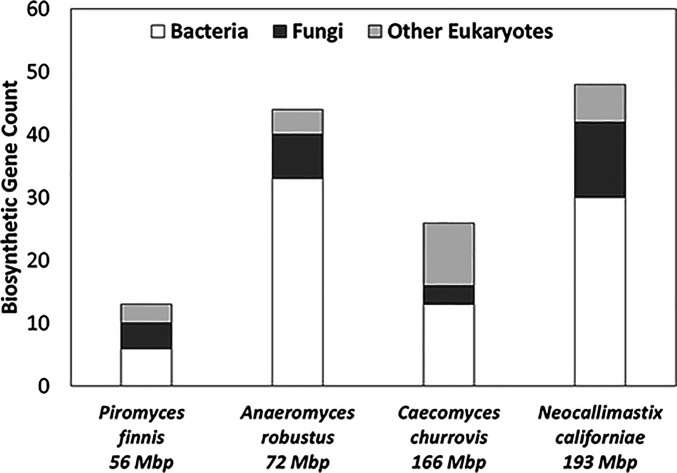
Biosynthetic genes from anaerobic fungi show the greatest similarity to bacteria. Core biosynthetic genes with at least three domains identified by antiSMASH were queried against NCBI’s nonredundant protein database using BLAST+. Top hits (largest bitscore) with E-value less than 1 × 10^−8^, greater than 30% identity, and greater than 25% coverage were classified for each biosynthetic gene according to taxonomy.

We compared the similarity of the genes encoding core biosynthetic enzymes to other organisms to deduce the novelty and phylogenetic origin of the genes. The top-scoring BLAST+ (41) hits (Fig. 2) for 20% of the total core biosynthetic genes were other fungal genes (protein sequence identity >30%, coverage >25%, and E-value <1 × 10^−8^). The majority of the homologous genes were hypothetical or uncharacterized proteins from other early-diverging fungi like chytrids, with a few instances of genes from higher-order fungi. A total of 80% of the homologous genes from higher-order fungi were from basidiomycetes, possibly due to the ancestral intake of basidiomycete fungi with forage by the herbivore hosts and subsequent horizontal gene transfer (HGT). However, the top hits for 63% of the gut fungal core biosynthetic genes appeared to be of bacterial origin rather than fungal.

Due to the high level of HGT associated the carbohydrate active enzymes of Neocallimastigomycetes (14), we probed whether any core biosynthetic genes may have also arisen in these fungi via HGT from bacteria. Phylogenetic trees of the PKS ketosynthase domains and NRPS condensations domains were constructed. These domains append additional subunits to the growing product chain, and they have been shown to be a good proxy for the entire biosynthetic gene when constructing phylogenies (42–45). HGT with bacteria was not supported for the PKS genes, since no ketosynthase domains nested within bacterial sequences and only 10% of domains were sister to bacteria. However, 44% of the fungal NRPS condensation domains were sister to or nested within domains from bacterial genes (*SI Appendix*, Fig. S1). Many of these bacteria are native to the rumen, such as *Clostridium cellulovorans*, thus supporting the hypothesis that some of the biosynthetic genes were likely horizontally transferred from bacteria. The majority of the transcribed NRPS genes were not part of a gene cluster (*SI Appendix*, Dataset S3). Therefore, at least some of the fungal NRPS genes may have been acquired by HGT of a single bacterial gene or transfer of an operon and subsequent loss of neighboring genes.

Similarly, phylogenies were constructed for the bacteriocins (*SI Appendix*, Figs. S2–S7). The putative bacteriocins were sister to Ciliophora, Firmicutes, or Actinobacteria (*SI Appendix*, Figs. S2 and S5–S7), with the exception of *C. churrovis* bacteriocins on scaffolds 90 and 616, which were most closely related to eukaryotes from Rhizaria (*SI Appendix*, Figs. S3 and S4). Therefore, the gut fungal bacteriocins may have been acquired from protozoa or bacteria in the rumen. There are many instances where HGT has been supported as the mechanism of acquisition of biosynthetic gene clusters (BGCs) between bacteria and fungi (43, 46–49) as well as between fungi (50), though such BGC HGT mechanisms have not been previously characterized in the rumen. However, the case for HGT is not as definitive for the bacteriocins as for the NRPSs, since in order to identify homologs, it was necessary to relax the E-value threshold to 0.1 and expand the search databases to include the Marine Microbial Eukaryote Transcriptome Sequencing Project (MMETSP) (51) (*Materials and* *Methods*). Nevertheless, it is clear that some of the genetic potential for natural products present in gut fungal genomes may be due to the complex microbial community in which they evolved.

Since 20% of the total biosynthetic genes were similar in sequence to other fungi, we investigated whether similarities existed in the regulation of secondary metabolism. Velvet regulatory proteins, which are characterized by a velvet domain ∼150 amino acids long, are known to coordinate development with secondary metabolism in other fungi, typically in complex with the methyltransferase LaeA and other velvet proteins (52, 53). Homologs of the developmental regulator *vosA* gene of *A. nidulans* (54, 55), which contains a velvet domain at the N terminus of the protein, were present in the *C. churrovis* (MycoCosm protein Ids 623244 and 624976), *N. californiae* (112212), and *P. finnis* (179530) genomes. These proteins have a primary region of homology centered at the velvet domain, with some conserved amino acids distal to the velvet domain (*SI Appendix*, Table S2 and Datasets S4–S6). Genes containing the velvet domain have been found in the genomes of other species of Chytridiomycota, such as the frog pathogen *Batrachochytrium dendrobatidis* (53). However, the anaerobic gut fungi are unique among the chytrids in that their genomes contain not only velvet homologs but also the biosynthetic machinery for secondary metabolism. Thus, the development and secondary metabolism of anaerobic gut fungi may be regulated by a velvet domain–containing protein acting in concert with other proteins. At present, it is not known whether the velvet proteins form a complex with a LaeA-like methyltransferase, similar to filamentous fungi.

### Polyketide Synthases Are Conserved between Genera of Anaerobic Gut Fungi.

**Fig. 3.**
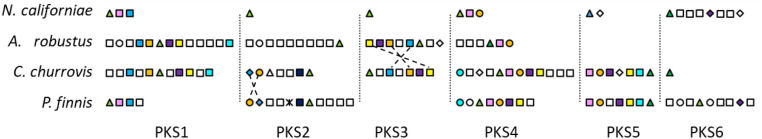
Many PKS families are conserved across genera of Neocallimastigomycetes. A total of 23 PKS genes predicted by antiSMASH across four fungal strains (vertical axis) can be represented in six PKS families (horizontal axis) as grouped by OrthoFinder (56). Only one member per species is depicted in the figure. Neighboring orthologous genes in each cluster are defined as bidirectional top-scoring BLASTp hits from filtered model proteins between genomes with E-value threshold of 10^−5^ and are indicated by matching colors in each PKS family. No color signifies the gene lacks a corresponding ortholog in the cluster. Full annotations of accessory genes are available in *SI Appendix*, Dataset S3. Triangle: PKS; Circle: posttranslational modification enzyme; asterisk: multiple genes depicted as a single gene; diamond: transporter; square: all other genes.

Although some of the core biosynthetic genes of anaerobic gut fungi are homologous to bacteria or higher fungi, the majority of PKSs are unique to the anaerobic gut fungi (*SI Appendix*, Dataset S7). On average, the PKS genes only share 34% amino acid identity to their top-scoring homolog, excluding Neocallimastigomycetes, and the highest similarity was only 39% (*C. churrovis* PKS on scaffold 118). We hypothesize that PKS genes present in multiple strains of gut fungi have important biological functions that confer fitness to anaerobic gut fungi, either by promoting their unique life cycle or distancing microbial competitors. A total of 23 iterative type I PKS genes of four or more enzymatic domains were identified by antiSMASH across all four fungal strains. These 23 PKS genes group into six PKS families by OrthoFinder (56). The complete OrthoFinder results, including additional genes identified as PKSs by SMURF, some of which were categorized as ClusterFinder fatty acids by antiSMASH, are presented in *SI Appendix*, Table S3. All of the families have antiSMASH-predicted genes in three or more strains, and PKS families 1, 2, and 4 are represented across all four strains. The corresponding gene clusters of the PKSs contain orthologous neighboring genes (Fig. 3 and *SI Appendix*, Dataset S3), which suggests that the polyketides in each family may serve a common function. A phylogenetic tree of the PKS genes of *A. robustus*, *C. churrovis*, *N. californiae*, and *P. finnis*, shown in *SI Appendix*, Fig. S8, affirms the close evolutionary relationships between the PKS genes of different fungal genera. All of the PKS genes are transcribed during the in vitro cultivation described previously (13, 57).

**Fig. 4.**
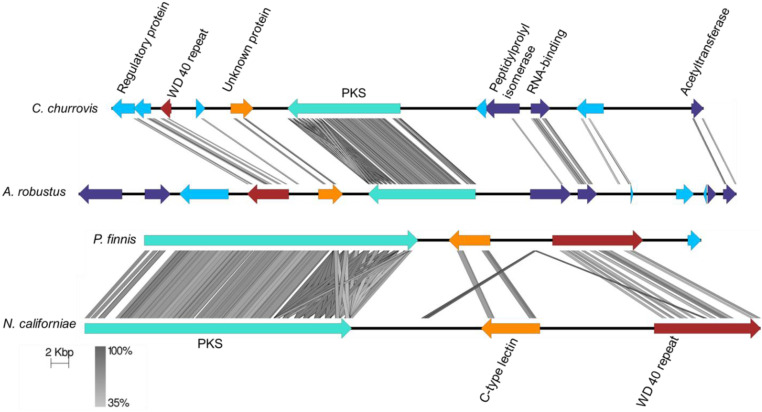
A PKS gene cluster is conserved among four strains of anaerobic gut fungi. Regions 50 bp and larger of at least 35% identity are highlighted in gray between genes. The turquoise PKS gene and red gene of unknown function encoding a protein with a WD 40 repeat are shared among all four strains. For a complete list of protein annotations, see *SI Appendix*, Dataset S3. Figure was generated using Easyfig (58).

To probe the conservation of neighboring genes in PKS gene clusters, we compared the PKS gene clusters from PKS family 1 (Fig. 4). Six genes were conserved between *C. churrovis* and *A. robustus*, and three genes were conserved between *N. californiae* and *P. finnis*. Across all four strains, the PKS gene and a gene of unknown function containing a WD-(trptophan-aspartic acid) 40 repeat were conserved. The PKS gene was present in two copies in the *N. californiae* genome: one copy on scaffold 26 and the second on scaffold 182. The gene cluster on scaffold 182 included Protein Id 705610, a Rap1-GTPase–activating protein, which was an ortholog of *A. robustus* Protein Id 283391. All constituent genes in these gene clusters were transcriptionally active. It is possible that these gene clusters constitute a polyketide biosynthesis pathway, but these gene clusters could also be an artifact of regions of conserved genomic synteny between fungal strains. Regardless of whether the PKS gene products independently synthesize a polyketide or are part of a more complex biosynthetic pathway, the conservation of the PKS gene suggests that the polyketide may be biologically important. One possibility is that the polyketide regulates the complex life cycle of anaerobic gut fungi. In the life cycle of anaerobic gut fungi, motile zoospores encyst into plant biomass and grow into a vegetative state, which develops reproductive sacs called sporangia that bear many zoospores (11). Secondary metabolites are known to regulate morphology and differentiation in other fungi, especially sporulation in ascomycetes (17).

### Transcriptomics, Proteomics, and N6-Methylation Indicate that Many of the Biosynthetic Genes of Anaerobic Gut Fungi Are Active during Standard Laboratory Cultivation.

**Fig. 5.**
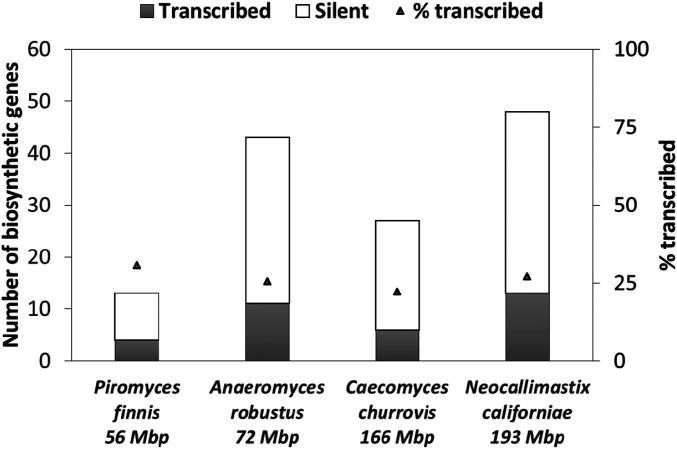
Many core biosynthetic genes of anaerobic gut fungi are transcribed during standard laboratory cultivation. Transcriptomes were previously acquired from anaerobic fungi cultivated on both grasses and soluble sugars (13, 57). The number of biosynthetic genes represented in the transcriptome is indicated by the gray bars, and the number of genes absent from the transcriptome (silent) are represented by empty bars. The percentage of transcribed genes is presented by the black triangles (secondary axis).

Following the establishment of the presence of biosynthetic genes in gut fungal genomes, we probed what proportion of these genes were expressed. We demonstrate here through a combination of transcriptomics, epigenetics, and proteomics that anaerobic gut fungi transcribe and translate a substantial portion of their core biosynthetic genes. Out of 131 total biosynthetic genes of three or more catalytic domains (e.g., adenylation) across all four fungal strains, 34 are actively transcribed at midlog phase during the standard laboratory growth conditions described previously (13, 57), whereas the remainder are silent (Fig. 5). The proportion of transcribed genes varied between 22 and 31% across all four strains of anaerobic gut fungi. Using five different media formulations with varied nutrient complexity and availability (*SI Appendix*, Table S4), 13 of the backbone genes of *N. californiae* were differentially regulated (*SI Appendix*, Fig. S9). The presence of messenger RNA (mRNA) and its regulation are promising indicators that some secondary metabolite genes are active even when anaerobic gut fungi are cultivated outside of their native environment. These results also support the recent finding by Amos and colleagues (59) that many biosynthetic genes are actively transcribed during midlog phase, not only during stationary phase. However, it is possible that more genes are expressed during late stationary phase, but this was not tested by transcriptomics due to prevalence of highly degraded mRNA from cultures harvested at that phase.

Another indicator of active genes in early-diverging fungi is the presence of adenine N6-methylation marks on the promoter regions (60). Dense methylated adenine clusters (MACs) were observed within 500 bp of the transcription start site of 6 out of 13 core biosynthetic genes for *P. finnis*, 8 out of 14 for *C. churrovis*, and 3 out of 46 core genes for *A. robustus* (*SI Appendix*, Dataset S8). In addition, neighboring genes in the gene clusters were also marked by MACs: 34 neighboring genes in *A. robustus* clusters, 33 in *P. finnis* clusters, and 43 in *C. churrovis* clusters. The Type 1 PKS genes identified by antiSMASH were highly methylated: five out of seven PKS genes from *P. finnis* and five out of six from *C. churrovis*. These data corroborate the transcriptomic evidence that anaerobic gut fungi actively transcribe a significant portion of their backbone genes and associated gene clusters during standard laboratory cultivation.

Finally, we searched for detectable proteins in both the membrane-bound and cytosolic fractions of fungal intracellular proteins. Proteomics confirmed that that at least 30% of the total biosynthetic enzymes from all four strains are translated into protein (*SI Appendix*, Table S5), thus increasing the likelihood that the secondary metabolism of anaerobic gut fungi is functionally active during laboratory cultivation. Notably, all copies of PKSs belonging to family 1 were expressed (*SI Appendix*, Table S5). In addition, PKS families 4 and 6, which were represented in the genomes of *C. churrovis*, *N. californiae*, and *P. finnis* were also expressed in these three strains. Across all lines of evidence (transcriptomics, N6-methylation, and proteomics), 53% of core biosynthetic genes were active by at least one metric.

### Three Groups of Natural Products from *A. robustus* and *N. californiae* Are Visualized via Molecular Networking of Tandem Mass Spectrometry Spectra.

**Fig. 6.**
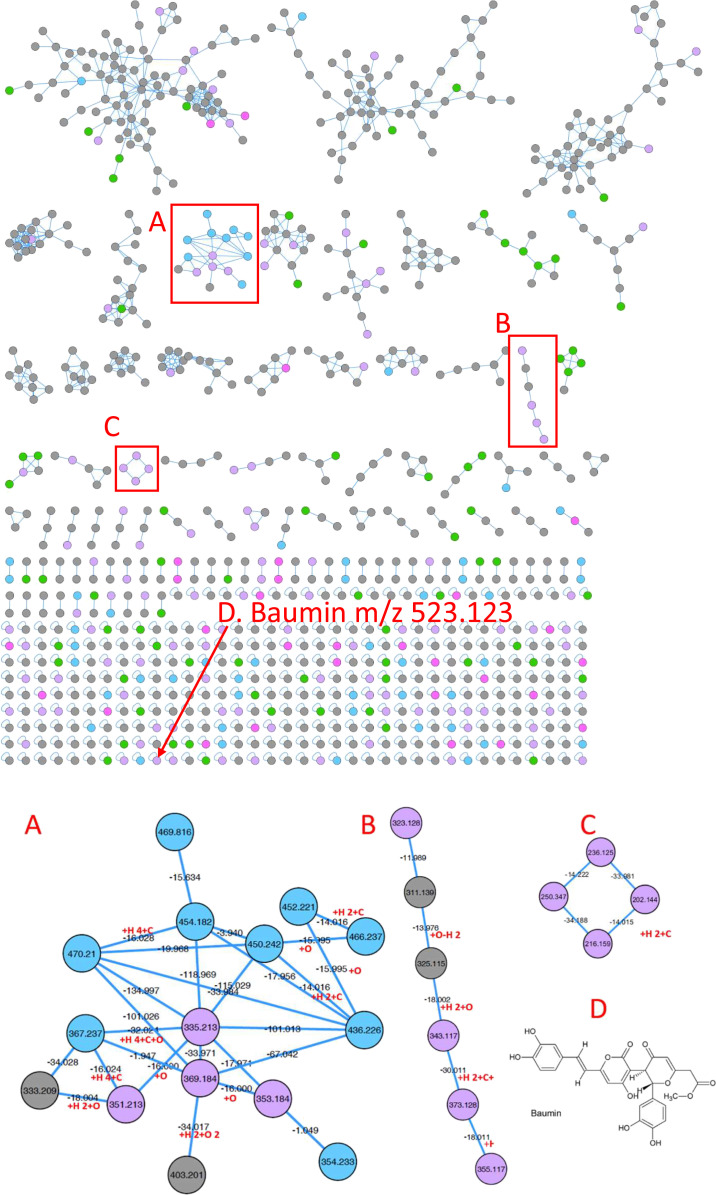
The molecular network generated from nonpolar untargeted metabolomics of *A. robustus* and *N. californiae* illustrates chemically diverse metabolites and natural products. Red rectangles enclose putative natural product clusters (*A*, *B*, and *C*) and baumin (*D*). Clusters *A*, *B*, and *C* are magnified below the network, and the chemical structure of baumin is shown in *D*. Node colors are as follows: blue = feature detected in *N. californiae* supernatant only, pink = *A. robustus* supernatant only, lilac = *A. robustus* and *N. californiae,* green = control only (autoclaved and incubated grass in liquid growth medium), gray = fungal supernatant and control. Self-looping nodes were truncated below baumin.

To further validate that anaerobic gut fungi synthesize natural products, we analyzed the nonpolar metabolites of *A. robustus*, *C. churrovis*, *N. californiae*, and *P. finnis* by liquid chromatography (LC)–tandem mass spectrometry (LC-MS/MS). We first built molecular networks using the Global Natural Products Social Molecular Networking (GNPS) platform (61) to distinguish groups of natural products based on LC-MS/MS datasets collected for *A. robustus* and *N. californiae*. To discriminate between compounds secreted by *A. robustus* or *N. californiae* and compounds already present in the complex growth medium or released from autoclaving the reed canary grass growth substrate, we constructed a molecular network showing separate conditions for secreted nonpolar metabolites from *A. robustus* or *N. californiae* and compounds from a control of complex growth medium. The majority of nodes in three clusters of the network (Fig. 6) were only present in the anaerobic fungal strains. None of the nodes matched the spectral libraries in GNPS. Similarly, we constructed a molecular network of the nonpolar metabolites of *C. churrovis* and *P. finnis* (*SI Appendix*, Datasets S9 and S10) and observed a cluster of 12 nodes present in the fungal supernatant but absent from the control and spectral libraries in GNPS. These findings support the hypothesis that the anaerobic gut fungi produce strain-specific as well as conserved secondary metabolites. Since the cultivation of anaerobic fungi requires the use of complex medium that contains a small amount of clarified rumen fluid, which is expected to harbor a low concentration of background secondary metabolites secreted by native microbes, there may be additional natural products that are present in both fungal supernatant and the growth medium.

### Anaerobic Gut Fungi Produce a Polyketide Related to the Antioxidant Baumin.

Among the 72 compounds detected from *A. robustus* (*SI Appendix*, Dataset S11), one had fragmentation spectra and exact mass consistent with the styrylpyrone baumin (*SI Appendix*, Dataset S12). Styrylpyrones are found in mushrooms, especially medicinal mushrooms, and are thought to have roles similar to those of flavonoids in plants, such as antioxidants. Baumin itself was first detected as a product of the fungus *Phellimus baumii* (now renamed *Sanghuangporus baumii*) from the distantly related fungal phylum Basidiomycota (62, 63). This compound, putatively identified as baumin, was also produced by *C. churrovis*, *N. californiae*, and *P. finnis.* In all strains, it was observed at 10-fold or greater intensity in the supernatant of fungal cultures compared to the growth medium (*SI Appendix*, Dataset S13).

We also used SIRIUS 4.0 (64) and CANOPUS (65) to predict the structure and class of the observed compound. Rather than baumin, SIRIUS predicted a flavonoid, whereas CANOPUS predicted a hydroxyflavonoid. However, upon inspection of the metabolic pathways annotated in the MycoCosm portal for *A.* *robustus*, we found that *A. robustus* lacked any genes encoding the biosynthetic enzymes of flavonoids. Furthermore, sequence alignment of flavonoid biosynthetic enzymes in higher-order fungi to the predicted proteins from *A. robustus* identified no homologs. Therefore, baumin remained the top candidate for the unknown compound. The putative baumin is a secondary metabolite directly detected from anaerobic gut fungi, and it may serve the anaerobic gut fungi as an oxygen scavenger in the rumen of the host animal after forage intake. Hobson reported up to 0.6% oxygen transiently present in the gaseous phase of the rumen (11, 66).

The gene cluster responsible for the production of baumin in *S. baumii* is not known at this time, which limited our ability to assign the gene cluster in anaerobic gut fungi. AntiSMASH predicted only one PKS gene cluster (accession OCB83923.1) with more than two domains from the *S. baumii* genome. The core biosynthetic gene was a hybrid NRPS-Type I PKS with PKS architecture of KS-AT-DH-KR-ACP (ketosynthase-acyltransferase-dehydratase-ketoreductase-acyl carrier protein). The domain architecture of this PKS is similar to PKS family 4 of the anaerobic fungi, although some members of this family lack the dehydratase domain (PKS genes from *A. robustus* scaffold 127, *C. churrovis* scaffold 129, and *N. californiae* scaffold 428). Protein BLAST results of the *S. baumii* PKS gene against the genes from PKS family 4 (*SI Appendix*, Table S3) are presented in *SI Appendix*, Datasets S14–S18. However, sequence similarity is only ∼30% between members of PKS family 4 from anaerobic fungi and the *S. baumii* PKS. Three additional PKS gene products (OCB90292.1, OCB89330.1, and OCB83944.1) were detected by a protein BLAST of the ketosynthase domain from OCB83923.1 against the National Center for Biotechnology Information (NCBI) nonredundant protein sequences database filtered by *S. baumii*. Using BLAST+ (41) in the MycoCosm portal (26), we queried the four PKS gene products from *S. baumii* against the filtered model proteins of each anaerobic gut fungus (*SI Appendix*, Table S6). Sequence alignments of OCB83923.1 resulted in the best combination of percent identity and subject coverage (>30% for both), but the corresponding subjects from each anaerobic gut fungus belonged to both PKS families 3 and 4. Based on these results, it remains unclear which gene cluster is responsible for baumin synthesis, and experimental validation is still necessary for both anaerobic gut fungi and *S. baumii*.

## Conclusion

Integrated 'omics' analysis of anaerobic gut fungi revealed the untapped potential of these nonmodel organisms as secondary metabolite producers. Species of anaerobic gut fungi from four distinct genera (*Anaeromyces*, *Caecomyces*, *Neocallimastix*, and *Piromyces*) possess the biosynthetic enzymes for polyketides, nonribosomal peptides, bacteriocins, and other natural product classes. The number of detected backbone genes per fungus is on the same order of magnitude as the natural product prolific aspergilli. Upon inspection, some of the biosynthetic genes of anaerobic fungi were similar to those found in bacteria, suggesting the possibility of horizontal gene transfer between fungi and bacteria in the rumen microbiome. HGT was further supported by the fact that in phylogenetic trees of NRPS condensations domains as well as bacteriocins, the fungal genes nested within or were sister to bacterial genes. Although many of the biosynthetic genes of anaerobic fungi were similar to bacteria, their regulation may still be typical of fungal secondary metabolism. Homologs of velvet regulatory proteins, which are known to link fungal development and secondary metabolism in filamentous fungi, were identified in the predicted proteins of anaerobic fungi. PKS genes identified within the fungal genomes were highly conserved between strains, indicating that polyketides may serve important biological functions for anaerobic fungi. Even during standard laboratory growth, transcriptomics and proteomics has demonstrated that much of their secondary metabolism is active. LC-MS/MS detected numerous secondary metabolites, including a compound putatively identified as the styrylpyrone baumin. Further experiments will be necessary to decipher the functions of the secondary metabolites of anaerobic fungi, but among many possibilities, they may serve as regulators of the fungal life cycle or defense or compounds against bacterial competitors. In addition to their native function, natural products from anaerobic gut fungi are a promising source of antimicrobial peptides, antibiotics, and therapeutics.

## Materials and Methods

### Routine Cultivation of Anaerobic Gut Fungi.

*A. robustus*, *C. churrovis*, and *N. californiae* were isolated via reed canary grass enrichment from the feces of sheep or goat at the Santa Barbara Zoo, as described previously (13, 14, 57). *P. finnis* was isolated from the feces of a horse at Verrill Farm Stables in Concord, MA, USA (13, 14, 57). The fungal strains were routinely transferred every 3 to 4 d into fresh reduced liquid medium with 0.1 g of 4 mm milled reed canary grass as growth substrate. *P. finnis* was cultivated in Medium C (67). *A. robustus*, *C. churrovis*, and *N. californiae* were cultivated in a reduced formulation of Medium C containing 0.25 g of yeast extract (Thermo Fisher Scientific), 0.5 g Bacto^TM^ Casitone, and 7.5% clarified rumen fluid.

### Mining Fungal Genomes for Biosynthetic Gene Clusters Using antiSMASH.

FASTA format genome files for *A. robustus* (14), *C. churrovis*, *N. californiae* (14), and *P. finnis* (14) (available from the MycoCosm (26) portal) were submitted separately to the antiSMASH 3.0 server (14, 60, 68). Default parameters were used, and the ClusterFinder analysis option selected. Since *C. churrovis* was sequenced after the launch of antiSMASH 4.0, it was analyzed by the legacy command line version of antiSMASH 3.0.

### Comparison of antiSMASH and SMURF-Based Biosynthetic Gene Predictions.

The command line implementation of BLAST+ (version 2.7.1) was used to construct a local database of the antiSMASH (68)-predicted biosynthetic genes and the MycoCosm (26) Secondary Metabolite (SM) Clusters (predicted from a SMURF (25)-derived algorithm). The antiSMASH library was queried against the SM Clusters library with the −gapopen and −gapextend options set to the maximum values (32767) to ensure gap-free alignments. The top bitscore hit for each query sequence were compiled into *SI Appendix*, Dataset S3.

### Protein BLAST Analysis of Core Biosynthetic Genes against NCBI Nonredundant Databases.

Core biosynthetic genes predicted by antiSMASH (19) were queried against the NCBI nonredundant protein databases using version 2.7.1 of the command line implementation of BLAST+ (41), excluding Neocallimastigomycota from the results. Thresholds for hits were as follows: minimum E-value 10^−8^, minimum qcovhsp 25%, and minimum identity 30%. The top hit for each query was considered to be the hit with the highest bitscore within these thresholds. The biosynthetic core gene in the reading frame with the most predicted domains was queried, except in cases where ClusterFinder (20) predicted multiple genes. In this case, all biosynthetic genes were searched. If the top hit for all genes was from the same taxonomic phylum, the result was counted once (e.g., cluster 3 on scaffold 152 of *N. californiae*). Otherwise, each phylum was counted. AntiSMASH genes containing fewer than three domains were not included in this analysis.

### Horizontal Gene Transfer Analysis of the PKS Ketosynthase Domains, NRPS Condensation Domains, and Bacteriocins.

PKS ketosynthase and NRPS condensation domains were selected based on Pfam (protein families) (69) annotations of protein sequences from the corresponding genomes. Selected sequences were used to search for homologs using BLAST+ (41) against NCBI’s nonredundant database (downloaded July 2019) with an E-value threshold of 1 × 10^−5^. Additionally, BLAST analysis was performed against fungal proteins from MycoCosm database (26), excluding sequences belonging to Neocallimastigomycota clade. For each ketosynthase domain, we constructed a phylogenetic tree based on selected homologs. We selected up to 10 best hits from 1) prokaryota, 2) nonfungal eukaryotes, and 3) non-Neocallimastigomycota fungal proteins, such that the maximum number of sequences used for each tree was 31. Sequences were aligned using MAFFT (Multiple Alignment using Fast Fourier Transform) (70) with subsequent removal of nonreliable aligned positions using trimAl (71). Phylogenetic trees were constructed using FastTree (72) and RAxML (73). We considered as potential nonfungal HGT events the cases when a given Neocallimastigomycota domain was nested within a prokaryotae or nonfungal eukaryota clade, and all branches of the tree had at least a 70% of bootstrap support values. Analysis of the bacteriocins was performed similarly, but the E-value threshold was relaxed to 0.1 and MMETSP (51) was queried in addition to MycoCosm and NCBI’s nonredundant databases.

### Identification of Velvet Homologs.

The velvet domain protein family has been previously established in filamentous fungi (53). The velvet domain family proteins were assigned to a Pfam (74), PF11754, as well as InterPro (75) family IPR021740. Filtered model proteins from of *A. robustus*, *C. churrovis*, *P. finnis*, and *N. californiae* belonging to PF11754 or IPR021740 were searched using the MycoCosm (26) portal. Two proteins were identified with the velvet motif in *C. churrovis*. Protein Id 623244 was a homolog of *vosA* from *A. nidulans* (accession ABI51618). Proteins were not identified in *A. robustus*, *P. finnis*, and *N. californiae* by Pfam domain search. To further expand the search, we used *vosA* from *A. nidulans* to query the other genomes by a protein BLAST+ search against all protein models in the remaining three strains of anaerobic gut fungi. Through this approach, we identified putative velvet proteins from *N. californiae* and *P. finnis* (*SI Appendix*, Table S2). The presence of a velvet domain was confirmed in all putative velvet proteins by CD-Search (76).

### Classification of Type I PKS Genes into Families and Assessment of PKS Gene Cluster Transcription.

PKS genes predicted by antiSMASH (19) and SMURF (25) for *A. robustus*, *C. churrovis*, *N*. *californiae*, and *P. finnis* were grouped into families by OrthoFinder (56) with default parameters. OrthoFinder defines an orthogroup as a group of genes that are descended from a common gene in the last common ancestor. The advantages of OrthoFinder include the ability to remove sequence length bias from sequence similarity scores as well as the ability to define orthogroup similarity limits (56). Further details about the OrthoFinder algorithm can be found in ref. 56. The OrthoFinder algorithm can be downloaded from www.stevekellylab.com/software/orthofinder. Transcriptionally active genes in the PKS gene clusters were determined using the metric of at least 5× coverage by RNA-seq reads over more than 95% of the length of the gene during standard laboratory cultivation described previously (13, 57).

### Curation of Biosynthetic Genes and Gene Clusters Using RNA-Seq Models.

All biosynthetic genes were manually curated to ensure that they are fully supported by RNA-seq data. In the case of incomplete models, we used the BRAKER1 pipeline (77), which combines usage of RNA-seq read alignments with GeneMark-ET and AUGUSTUS gene finding to extend the gene models to completeness. RNA-seq data were previously acquired from fungal cultures grown on grasses and soluble sugars (13, 57). Clusters were delineated starting with the core biosynthetic gene and determining all genes in the 5′ and 3′ direction with at least 5× coverage across at least 95% of the gene length by RNA-seq reads and within 10 kbp consecutive intergenic distance of their neighbor.

### Quantification of Biosynthetic Gene Transcription and N6-Methylation.

Transcriptome assemblies for *A. robustus*, *C. churrovis*, *N. californiae*, and *P. finnis* were described previously (13, 57). To determine the number of transcribed biosynthetic genes, local BLAST libraries were prepared from the antiSMASH amino acid predictions of biosynthetic genes (e.g., PKS and NRPS). A protein BLAST was performed locally using Blast2GO (78) with the respective transcriptome as the subject sequences (78). The biosynthetic genes were considered transcribed if hits were returned with E-value cutoff 0.001, similarity greater than 95%, and Hsp/Query greater than 95%. Only antiSMASH genes containing three or more catalytic domains (e.g., adenylation) with at least 100 amino acids were queried.

N6-methyldeoxyadenine positions were collected either from previously published data (60) (*P. finnis* and *A. robustus*) or using the Sequel I PacBio sequencing platform (*C. churrovis*) followed by analysis using the smrtlink version 8.0.0.80529 pb basemods workflow. Following detection, 6 mA modified sites were filtered and MACs were identified as described previously (60). Dense MACs were quantified as described previously (60). Promoters were considered methylated if MACs were present within 500 bp of the transcription start site.

### Sample Preparation for Proteomics.

Anaerobic serum bottles containing 40 mL of Medium C (67) and 5 g/L cellobiose (Fisher Scientific) were preheated to 39 °C and then inoculated with 1.0 mL each of *A. robustus*, *C. churrovis*, *N. californiae*, or *P. finnis* from routine passaging. After 3 d of growth (4 d for *P. finnis*), 1.0 mL of each culture was used to inoculate each serum bottle for later proteomic analysis. Each serum bottle contained 80 mL of Medium C with 5 g/L cellobiose (Fischer Scientific) as the carbon source. For each fungal strain, six 80 mL cultures were prepared. After 3 d of growth, *C. churrovis* cultures were harvested, and after 6 d of growth, cultures of *A. robustus*, *N. californiae*, and *P. finnis* were harvested. The cultures were transferred into 50 mL Falcon tubes and centrifuged at 3,200 *g* and 4 °C for 10 min using a swinging bucket rotor (Eppendorf A-4-81). The supernatant was removed, and each pellet was washed with 5.0 mL of pH 7.4 phosphate-buffered saline (PBS) solution and centrifuged again to remove the PBS. Samples were frozen at −80 °C until the time of extraction.

For proteomic extraction, all chemicals were obtained from Sigma Aldrich unless otherwise noted. Fungal cell pellets were extracted utilizing a method similar to MPLEx (79), where the pellets were suspended in 5 mL ice-cold water, 6.75 mL methanol, and homogenized with a disposable probe homogenizer (Omni International). Ice-cold chloroform was then added to the homogenate so that the chloroform:methanol:water ratio was 8:4:3, and the samples were vigorously vortexed for 1 min. The samples were placed on ice for 5 min, followed by another 1 min vortex step. The samples were then centrifuged at 5,000 *g*, 4 °C for 10 min. The top (polar metabolite) layer of the triphasic separation and the lower (nonpolar metabolite) layer were not used in this study. The protein interphase pellet was washed with 1 mL of ice-cold methanol by vortexing and centrifuging as above. The supernatant was removed and disposed, and the pellets allowed to dry slightly. The pellets were reconstituted in an 8 M urea, 100 mM NH_4_HCO_3_ buffer, and a Bicinchoninic Acid protein assay (Thermo Fisher Pierce) was performed to quantify the protein content in the pellet. A total of 1 mg of protein was utilized for digestion, normalized to the same volume for all samples. Dithiothreitol was added to a 5 mM concentration in each sample, and the samples were incubated at 37 °C for 1 h with shaking at 800 rpm on a thermomixer (Eppendorf). The samples were then diluted eightfold with 50 mM NH_4_HCO_3_, and calcium chloride was added to a concentration of 1 mM. Trypsin was added in a 1:50 (w:w) trypsin:protein ratio and samples were incubated for 3 h at 37 °C. Samples were centrifuged at 5,000 *g* at room temperature for 10 min to pellet particulate material, and the clarified samples were then subjected to C18 solid phase extraction cleanup as described previously (14) with the exception that a Strata C18-E 50 mg column (Phenomenex) was used. The samples were then diluted to a concentration of 0.1 μg/uL for LC-MS analysis.

### Proteomics Mass Spectrometry and Data Analysis.

Separation was performed prior to mass spectrometry (MS) by a Waters nanoAcquity M-Class dual pumping ultra-performance liquid chromatography system (UPLC) using a 5 µL injection at 3 µL/min with reverse-flow elution onto the analytical column at 300 nL/min. The gradient profile of mobile phases of A) 0.1% formic acid in water, and B) 0.1% formic acid in acetonitrile was the following (min, %B): 0, 1; 2, 8; 20, 12; 75, 30; 97, 45; 100, 95; 110, 95; 115, 1; and 150, 1. The trapping column was 150 µm internal diameter (ID) and 4 cm in length, and the analytical column was 75 µm ID and 70 cm in length. The columns were packed in-house using 360 µm outer diameter (OD) fused silica (Polymicro Technologies Inc.) with 2 mm sol-gel frits for media retention and contained Jupiter C18 media (Phenomenex). Particle sizes for the trapping and analytical columns were 5 and 3 µm, respectively.

Proteomics data were collected on a Q-Exactive Plus mass spectrometer (Thermo Scientific) with a homemade nano-electrospray ionization interface. The electrospray emitters were prepared from 150 μm OD × 20 μm ID chemically etched fused silica (80). The spray voltage was 2.2 kV, and the ion transfer tube temperature was 250 °C. Data were collected for 120 min after a 20 min delay from time of sample injection and trapping. Fourier transform- (FT) MS spectra were acquired with a resolution of 35 k (AGC target 3 × 10^6^) from 400 to 2,000 *m*/*z*. The top 12 FT-HCD-MS/MS spectra were acquired in data-dependent mode, excluding singly charged ions, with a resolution of 17.5 k (AGC target 1 × 10^5^) and an isolation window of 2.0 *m*/*z* using a normalized collision energy of 30 and exclusion time of 30 s.

Proteomics data analysis was performed as previously described (14), with the exception that peptide fragments were mapped to the transcriptomes (13, 57) translated into all open reading frames as well as to the antiSMASH-predicted biosynthetic enzymes.

### Preparation of Fungal Supernatant Samples for Metabolomics.

Routinely passaged *A. robustus*, *C. churrovis*, *N. californiae*, or *P. finnis* was inoculated into 60 mL serum bottles (VWR International) containing 40 mL of anaerobic, autoclaved Medium C (67) with 0.4 g of 4 mm-milled reed canary grass. After 3 d of growth at 39 °C, these seed cultures were used to inoculate four replicate Hungate tubes per fungal strain containing 9 mL of anaerobic, autoclaved Medium C and 0.1 g of 4 mm-milled reed canary grass. For each serum bottle or Hungate tube, 1.0 mL of fungal inoculum was used. Four Hungate tubes containing anaerobic, autoclaved Medium C and reed canary grass were incubated at 39 °C for use as a control. Cultures and controls were harvested after 6 d of incubation, centrifuged at 3,220 *g* for 10 min with the swinging bucket rotor (Eppendorf F-34-6-38) at 4 °C, and the fungal supernatant was frozen at −80 °C for exometabolomics analysis.

### Extraction and LC-MS/MS of Nonpolar Metabolites.

Ethyl acetate extraction of nonpolar metabolites from fungal supernatant was performed as follows: 2 mL ethyl acetate was added to 1.5 to 2 mL fungal supernatant, vortexed and sonicated for 10 min in a water bath (room temperature), centrifuged (5 min at 5,000 rpm), then the top ethyl acetate layer removed to another tube. For 2 mL supernatant samples, this process was repeated with another 2 mL ethyl acetate added to the sample, then the top layer removed and combined with the previous extract. Extracts were dried in a SpeedVac (SPD111V, Thermo Scientific) and stored at −20 °C.

In preparation for LC-MS analysis, 150 to 300 µL LC-MS grade methanol containing 1 µg/mL internal standard (2-Amino-3-bromo-5-methylbenzoic acid, Sigma) was added to dried extracts, followed by a brief vortex and sonication in a water bath for 10 min, then centrifugation for 5 min at 5,000 rpm. A total of 150 µL of resuspended extract was then centrifuge filtered (2.5 min at 2,500 rpm) using a 0.22 μm filter (UFC40GV0S, Millipore) and transferred to a glass autosampler vial. Reverse phase chromatography was performed by injecting 2 μL of sample into a C18 chromatography column (Agilent ZORBAX Eclipse Plus C18, 2.1 × 50 mm 1.8 µm) warmed to 60 °C with a flow rate of 0.4 mL/min equilibrated with 100% buffer A (100% LC-MS water with 0.1% formic acid) for 1 min, followed by a linear gradient to 100% buffer B (100% acetonitrile with 0.1% formic acid) at 7 min, and then held at 100% B for 1.5 min. MS and MS/MS data were collected in centroid form in both positive and negative ion mode using a Thermo Q Exactive HF mass spectrometer (ThermoFisher Scientific) with full MS spectra acquired ranging from 80 to 1,200 *m*/*z* at 60,000 resolution, and fragmentation data was acquired using an average of stepped collision energies of 10, 20, and 40 eV at 17,500 resolution and 20, 50, and 60 eV for a single replicate. Mass spectrometer source settings included a sheath gas flow rate of 55 (au), auxiliary gas flow of 20 (au), sweep gas flow of 2 (au), spray voltage of 3 kV, and capillary temperature of 400 °C. Sample injection order was randomized, and an injection blank of methanol only run between each sample. Raw data are available for download at https://genome.jgi.doe.gov/portal/.

### Metabolomics Data Analysis and Identification of Baumin from *A. robustus.*

Peak finding was performed with MZmine (81). Filtering the features that were at least fourfold higher in intensity than the control resulted in 116 peaks. Metabolite Atlas workflow tools were used to remove features that were visually recognized as artifacts of the peak-finding process (isotopes, background ions, unnecessary adducts, etc.) and to refine retention times for feature integration across files (82). These features were further filtered to those with sample/control ratios of at least 4.

Each MS2 fragmentation spectrum was searched with Pactolus, an in-house implementation of the MIDAS (Metabolite Identification via Database Searching) (83) scoring algorithm. The MIDAS scoring algorithm traverses the possible fragmentation paths and scores an identification based on *m*/*z* values matching structures along a fragmentation path. The Pactolus implementation precomputes and stores the fragmentation paths for each molecule, whereas the MIDAS algorithm computes the fragmentation paths on the fly. The Pactolus code can be found at https://github.com/biorack/pactolus. Suggested identifications for each MS/MS spectrum were mapped back to the filtered list of features above. For the feature *m*/*z* 523.1232 at 4.7 min, the compound baumin was identified as a top hit as calculated by Pactolus. To investigate the likelihood of this identification, an isotope simulation using XCalibur 2.2SP1 (Thermo Scientific) was used to determine potential molecular formulas of the feature. The chemical formula C27H22O11, which corresponds to baumin, was calculated as a protonated adduct with an error of −0.288 ppm. Further support for this identification is given by the MS/MS spectra, with detected *m*/*z* supporting likely fragments of the baumin structure (*SI Appendix*, Dataset S12). Peak finding was performed as above for *C. churrovis*, *N. californiae*, and *P. finnis* to ascertain whether baumin was also produced by those strains.

The MS-GF+ (1.3.0) Workflow was used in the ProteoSAFe web server, available through the Center for Computational Mass Spectrometry, to run structure and class prediction by SIRIUS 4.0 (64) and CANOPUS (65), respectively. The job is publicly available at the following URL: https://proteomics2.ucsd.edu/ProteoSAFe/status.jsp?task=6e6cf05848424ca1bdba0f5b48ccafc6

We checked *A. robustus* for its genetic capability to produce flavonoids. No gene models were assigned to the Flavonoid Biosynthesis KEGG (84) map, which is a component of MAP01060 on the MycoCosm portal (26). The following genes related to flavonoid biosynthesis in higher-order fungi, selected from *SI Appendix*, Supplementary File S1 of ref. 85, were queried using BLAST+ (41) against filtered model proteins of *A. robustus* in the MycoCosm portal (26): RAQ52167.1 (chalcone synthase, *Aspergillus flavus*), PYH41479.1 (chalcone isomerase, *Aspergillus saccharolyticus*), PIG83686.1 (dihydroflavonal-4-reductase, partial, *Aspergillus arachidicola*), CEL03464.1 (Isoflavone reductase family protein, *Aspergillus calidoustus*), RAH85834.1 (quercetin 2,3-dioxygenase anaerobically complexed with the substrate kaempferol, *Aspergillus japonicus* CBS 114.51), RAQ53266.1 (leucoanthocyanidin dioxygenase, *Aspergillus flavus*), GAO86351.1 (myricetin O-methyltransferase, *Aspergillus udagawae*), CCE28660.1 (related to naringenin, 2-oxoglutarate 3-dioxygenase, *Claviceps purpurea*), and 20.1, RAQ48556.1 (quercetin 2, *Aspergillus flavus*). There were no hits for any of these sequences (E-value threshold 10^−5^).

### Visualization of Nonpolar Untargeted Metabolomics Data via Molecular Networking.

Molecular networks were constructed from the MS/MS data described above. Two separate networks were constructed: one for *A. robustus* and *N. californiae* and a Medium C control and the other for *C. churrovis*, *P. finnis*, and a Medium C control. Molecular networks were created using the online workflow at GNPS (61). All MS/MS peaks within ^+/−^ 17 Da of the precursor *m*/*z* were removed. The MS/MS spectra were further filtered by selecting only the top six peaks in the ^+/−^ 50 Da window throughout the spectrum. Consensus spectra were created by clustering the data using MS-Cluster (86) with a MS/MS fragment ion tolerance of 0.5 Da and a parent mass tolerance of 2.0 Da. Consensus spectra with fewer than two spectra were eliminated. A network was then created with the following criteria used for the edges: 1) cosine score above 0.7 and 2) greater than six matched peaks. Furthermore, edges between nodes were only considered in the network if and only if each of the consensus spectra represented by the nodes were part of other node’s top 10 most similar nodes. The spectra in the network were subsequently queried against the spectral libraries of GNPS. The library spectra were filtered to be consistent with the input data. Only pairings between network spectra and library spectra with scores above 0.7 and at least six matched peaks were kept.

## Supplementary Material

Supplementary File

Supplementary File

Supplementary File

Supplementary File

Supplementary File

Supplementary File

Supplementary File

Supplementary File

Supplementary File

Supplementary File

Supplementary File

Supplementary File

Supplementary File

Supplementary File

Supplementary File

Supplementary File

Supplementary File

Supplementary File

Supplementary File

## Data Availability

The MS data were deposited in public repository MassIVE (MSV000085907). The GNPS jobs for *A. robustus* and *N. californiae* can be accessed at https://gnps.ucsd.edu/ProteoSAFe/status.jsp?task=af74716b912a435eb53c1307a1dad092. The GNPS job for *C. churrovis* and *P. finnis* can be accessed at https://gnps.ucsd.edu/ProteoSAFe/status.jsp?task=6fb6a9367cf34b669b8bc00862541af9. AntiSMASH GenBank files of the core biosynthetic genes identified in the genomes of *A. robustus*, *C. churrovis*, *N. californiae*, and *P. finnis* are available at the following GitHub repository: https://github.com/cswift3/Additional-datasets-for-Anaerobic-fungi-are-an-untapped-reservoir-of-natural-products. This repository also contains phylogenetic trees in Newick format supporting the HGT analysis of NRPS condensation domains and PKS ketosynthase domains of *A. robustus*, *C. churrovis*, *N. californiae*, and *P. finnis*. Last, this repository contains molecular networks corresponding to Fig. 6 and *SI Appendix*, Dataset S9 in GRAPHML format for visualization in Cytoscape (87). All other study data are included in the article and/or supporting information.
