# Supplementary material for: Anaerobic gut fungi are an untapped reservoir of natural products
Source: Proc Natl Acad Sci U S A. 2021 Apr 27;118(18):e2019855118. doi: 10.1073/pnas.2019855118 (PMC8106346; doi:10.1073/pnas.2019855118)
Supplement: Supplementary File [file pnas.2019855118.sapp.pdf]

PNAS
www.pnas.org

Supplementary Information for

Anaerobic gut fungi are an untapped reservoir of natural products

Candice L. Swift, Katherine B. Louie, Benjamin P. Bowen, Heather M. Brewer, Samuel O. Purvine, Asaf Salamov, Stephen J. Mondo, Kevin V. Solomon, Aaron T. Wright, Trent R. Northen, Igor V. Grigoriev, Nancy P. Keller, Michelle A. O'Malley*

*Email: momalley@engineering.ucsb.edu

This PDF file includes:

Supplementary text
Supplementary methods
Figs. S1 to S9
Tables S1 to S6
Captions for datasets S1 to S18
References for SI reference citations

Other supplementary materials for this manuscript include the following:

Datasets S1 to S18

Supplementary text

Validation of predicted bacteriocins

APD3 (1) identified two of the fungal putative bacteriocins as candidate AMPs: *C. churrovis* cluster 6 on scaffold 90, likely an α-helical AMP, and *N. californiae* cluster 4 on scaffold 363. *C. churrovis* cluster 23 has a charge of -6. Since bacteriocins are typically positively charge (1), *C. churrovis* cluster 23 is unlikely to be a true bacteriocin.. The remaining sequences identified as bacteriocins by antiSMASH (2) were longer than 200 amino acids and thus outside the scope of the calculator.

Comparison of antiSMASH vs. SMURF predicted core biosynthetic genes

SMURF-based predictions are available in the MycoCosm portal (3) Secondary Metabolism Clusters. In some cases, antiSMASH predicted biosynthetic genes in different reading frames as part of the same gene cluster. The *A. robustus* NRPS gene in cluster 23 located on scaffold 211 corresponded to two different SMURF predictions. The longest reading frame (ctg211_allorf000654) matched Cluster Id Anasp1.33 and another containing adenylation and ketosynthase domains (ctg211_allorf000784) matched Anasp 1.34. In other cases, the two algorithms identified the same gene but predicted different natural product classes. For example, *N. californiae* cluster 15 located on scaffold 137 is a ClusterFinder fatty acid predicted by antiSMASH, whereas it is considered a PKS by SMURF (Neosp1.12). This is not surprising considering that many of the PKS genes of anaerobic gut fungi are highly reduced. In one instance, antiSMASH combined a ClusterFinder fatty acid gene and an NRPS into a single, hybrid cluster (cluster 4 located on *N. californiae* scaffold 161), whereas the SMURF-based algorithm separated these clusters into a PKS (Neosp1.37) and an NRPS (Neosp1.38). Despite these differences, 90% of the backbone genes predicted by SMURF in each fungal strain were located on scaffold regions the same as or overlapping with backbone genes predicted by antiSMASH. The following scaffolds predicted by SMURF to harbor biosynthetic genes were not identified by antiSMASH: *P. finnis* scaffold 1 (Pirfi3.6), *C. churrovis* scaffold 143 (Caecom1.3), *N. californiae* scaffold 146 (Neosp1.21), *N. californiae* scaffold 59 (Neosp1.24), *N. californiae* scaffold 58 (Neosp1.26), *N. californiae* scaffold 1616 (Neosp1.34), *A. robustus* scaffold 582 (Anasp1.6), *A. robustus* scaffold 24 (Anasp1.8), *A. robustus* scaffold 291 (Anasp1.17), *A. robustus* scaffold 197 (Anasp1.36), and *A. robustus* scaffold 207 (Anasp1.45).

Supplementary methods

RNA extraction, sequencing, and differential expression analysis of *N. californiae* grown in media formulations of varying nutrient availability and complexity

N. californiae was grown in five different media formulation varying in nutrient availability and complexity (Table S4) until early stationary phase, as assessed by the accumulated pressure method (4). Cultures were grown in quadruplicate Hungate tubes, with each tube containing 9 mL of each media formulation and 0.1 g milled reed canary grass as the carbon source. All liquid media were prepared using the same batch of rumen fluid for consistency. The seed culture used to inoculate all replicates was started from 2.0 mL of *N. californiae* (from routine passaging) inoculated into a serum bottle containing 115 mL of medium M2 (5) with 1.1 g milled reed canary grass. Prior to inoculation, the serum bottle, Hungate tubes, and their contents were sparged with CO_2 and autoclaved. Vitamin solution (5) was added to the tubes and bottle after autoclaving to a final concentration of 1 vol%, and the tubes and bottle were pre-warmed to 39 °C. 1.0 mL of the seed culture was inoculated into each replicate Hungate tube.

Once cultures reached early stationary phase (between 30 h and 96 h of growth, depending on the media formulation) Cultures were transferred to 15 mL Falcon™ tubes (Fisher

Scientific) and centrifuged at 4 °C and 12,000 *g* with a fixed angle rotor (Eppendorf™ F-34-6-38) for 10 min. Supernatant was removed by pipette and 1.0 mL of RNAlater® (Sigma-Aldrich) was added to each Falcon™ tube.

Samples were thawed on ice, centrifuged at 12,000 *g* for 10 min using a fixed angle rotor (Eppendorf™ F-34-6-38) at 4 °C, and the supernatant (RNAlater®) was removed by pipette. The pellet was transferred by a RNAse-free spatula to autoclaved 2 mL screw-cap tubes containing 1.0 mL of 0.5 mm zirconia/silica beads (Biospec) and 450 µL of buffer RLT (QIAGEN) with 1 vol% 2-mercaptoethanol (Sigma-Aldrich). The fungal cells were then lysed by bead-beating for 1 min using a Biospec Mini-beadbeater-16. Following lysis, the tubes were placed on ice for 30 s. Subsequently, the tubes were centrifuged for 3 min at 13,000 g and 22 °C with a microcentrifuge (Eppendorf™ 5424). To maximize yield, gel loading tips (Fisher Scientific) were used to remove the supernatant, which was transferred to round-bottom sample tubes (QIAGEN catalog number 990381). Total RNA was extracted by QIAcube following the RNeasy Mini protocol for animal cells with QIAshredder homogenizenization and optional on-column DNase digest.

RNA quantity was measured by an Invitrogen Qubit 2.0 fluorometer and quality was assessed by TapeStation (Agilent). All RNA had an RNA Integrity Number (RIN) greater than 7.5, except for one sample with RIN 6.2. The mRNA library was prepared for sequencing using the Illumina® Truseq® Stranded mRNA kit, which selects for eukaryotic polyadenylated mRNA using poly-T beads. An Illumina® NextSeq500 sequenced the library with greater than 400 million 75 bp single-end reads. The reads were aligned to the previously assembled transcriptome of *N. californiae* (6) using RSEM (7). Differential expression analysis was performed by DESeq2 in R (8).

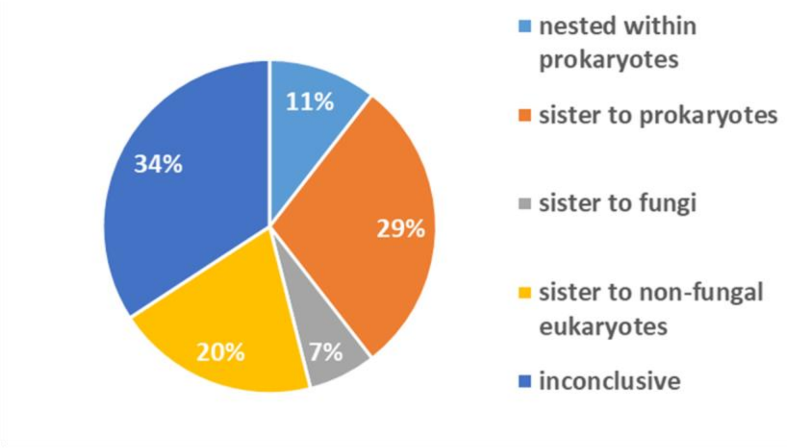

Fig. S1. Anaerobic gut fungi share NRPS condensation domains with prokaryotic community members. Pie chart showing the phylogenetic relationships between NRPS condensation domains of *A. robustus*, *C. churrovis*, *N. californiae*, and *P. finnis* and homologs identified using BLAST+ (9) against NCBI non-redundant databases. Phylogenetic trees were constructed using FastTree (10).

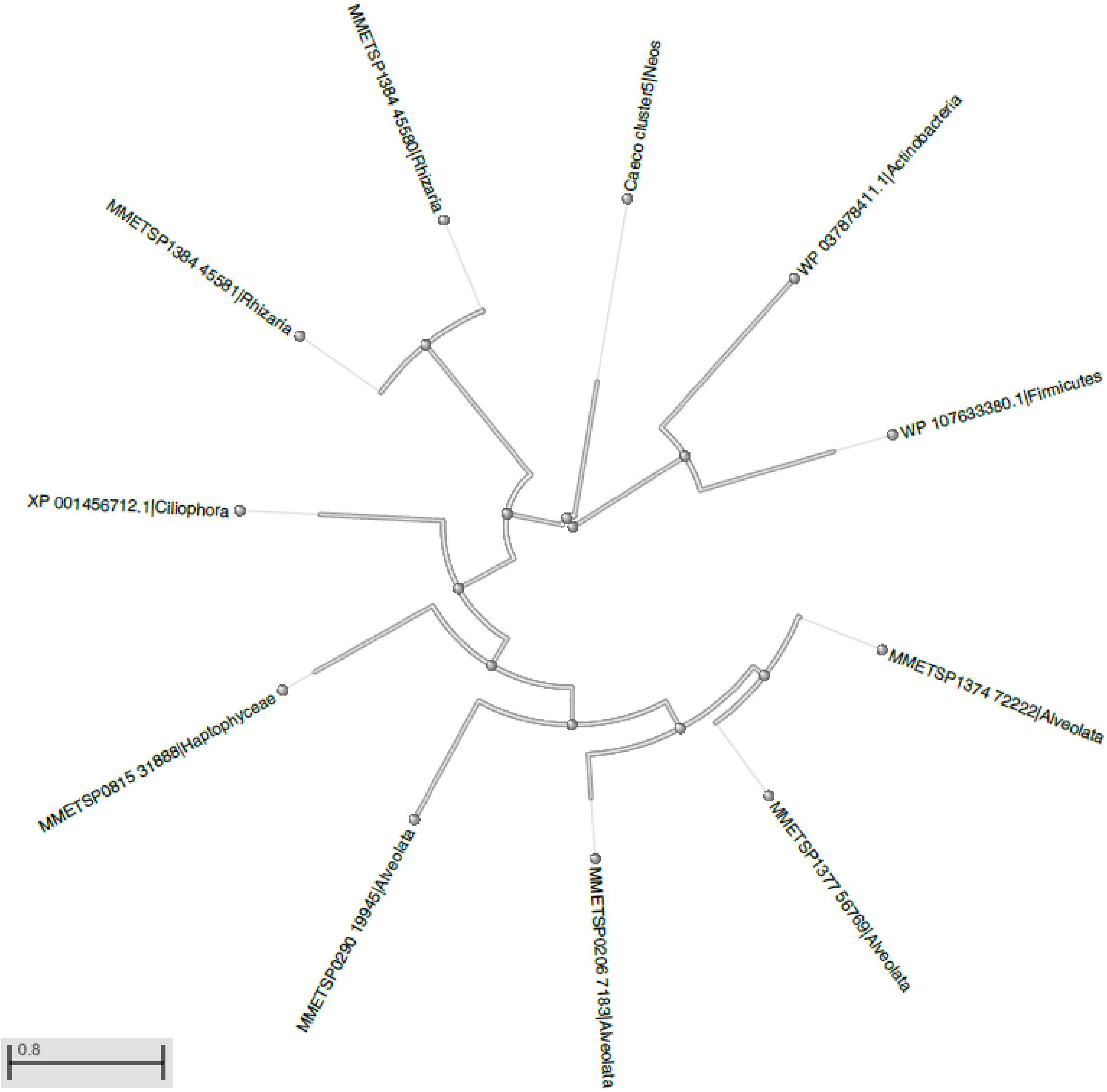

Fig. S2. Phylogeny of *C. churrovis* bacteriocin located on scaffold 83 (antiSMASH cluster 5), represented as "Caeco cluster 5|Neos." Tree rendered using NCBI Tree Viewer 1.17.5.

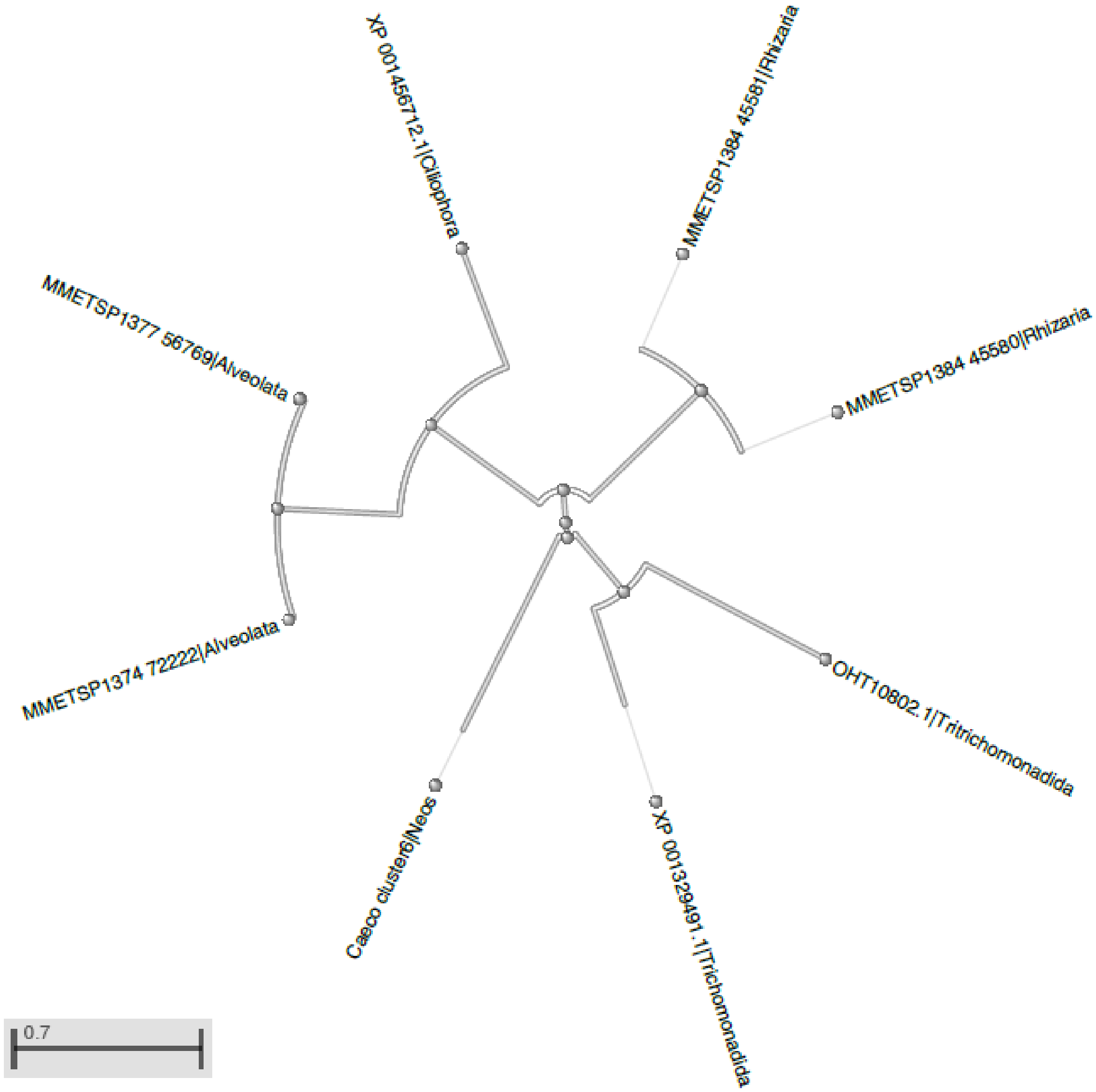

Fig. S3. Phylogeny of *C. churrovis* bacteriocin located on scaffold 90 (antiSMASH cluster 6), represented as "Caeco cluster 6|Neos." Tree rendered using NCBI Tree Viewer 1.17.5.

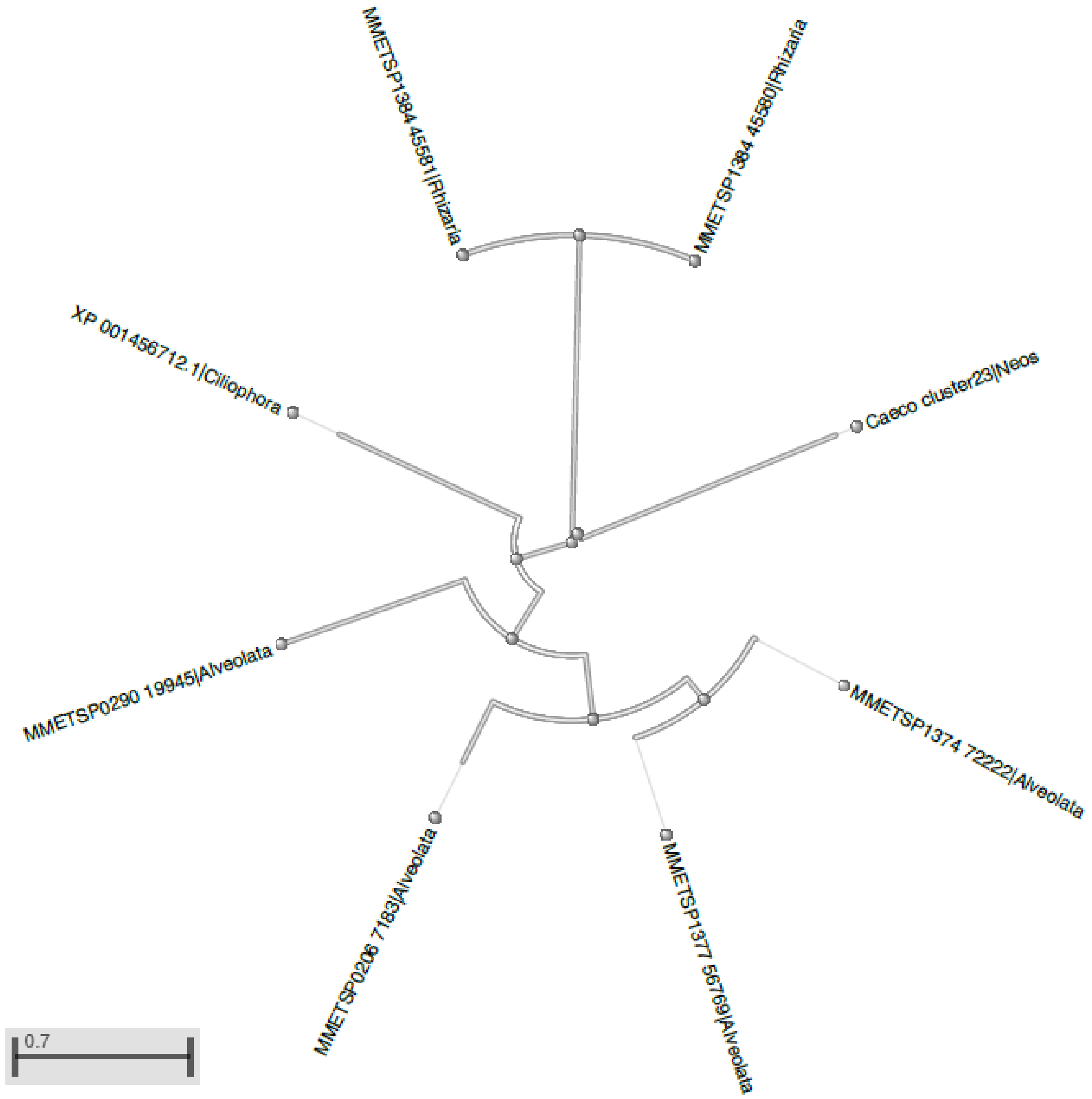

Fig. S4. Phylogeny of *C. churrovis* bacteriocin located on scaffold 616 (antiSMASH cluster 23), represented as "Caeco cluster23|Neos." Tree rendered using NCBI Tree Viewer 1.17.5.

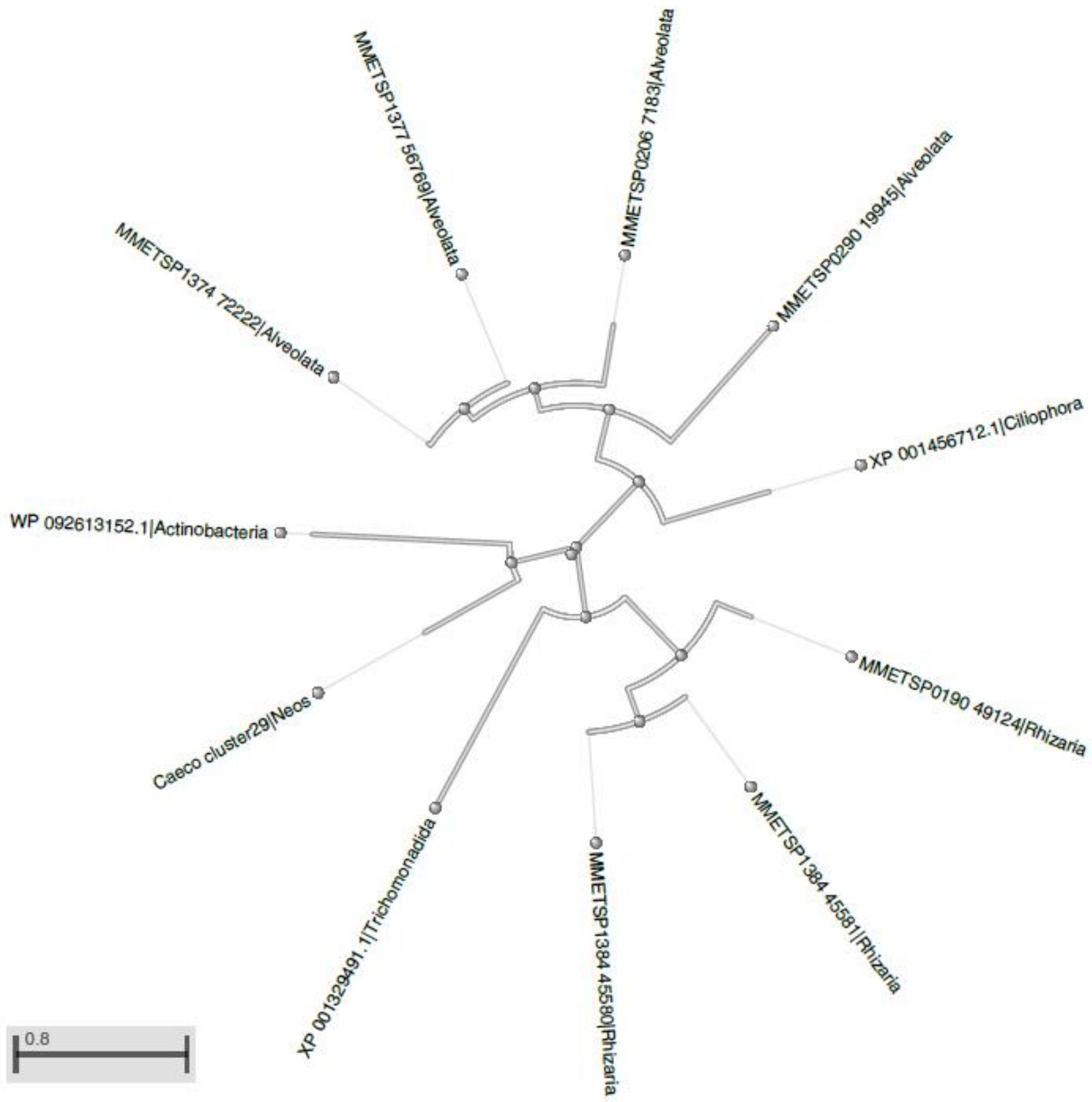

Fig. S5. Phylogeny of *C. churrovis* bacteriocin located on scaffold 1501 (antiSMASH cluster 29), represented as "Caeco cluster 29|Neos." Tree rendered using NCBI Tree Viewer 1.17.5.

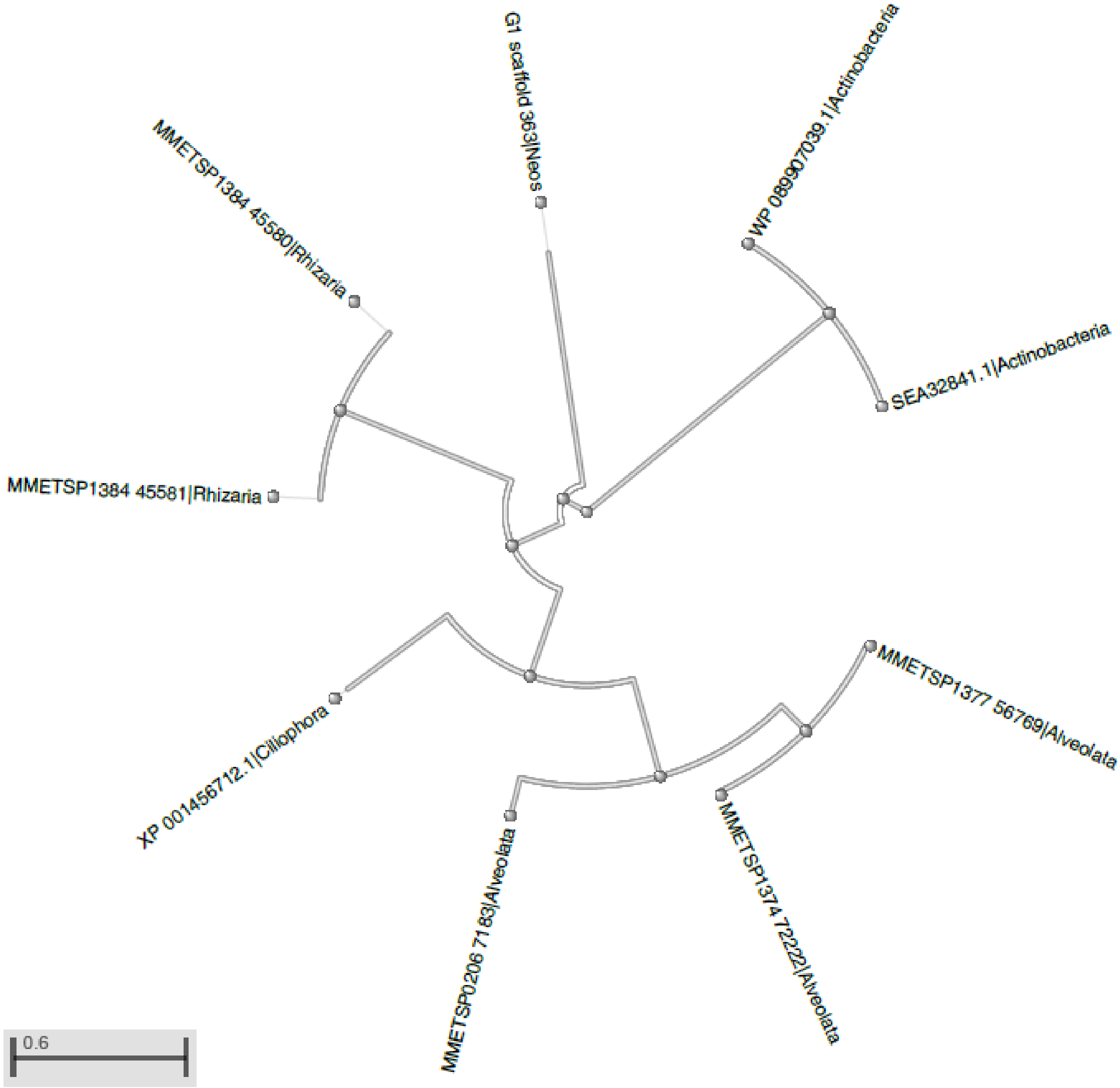

Fig. S6. Phylogeny of *N. californiae* bacteriocin located on scaffold 363 (antiSMASH cluster 4), represented as G1 scaffold 363|Neos.” Tree rendered using NCBI Tree Viewer 1.17.5.

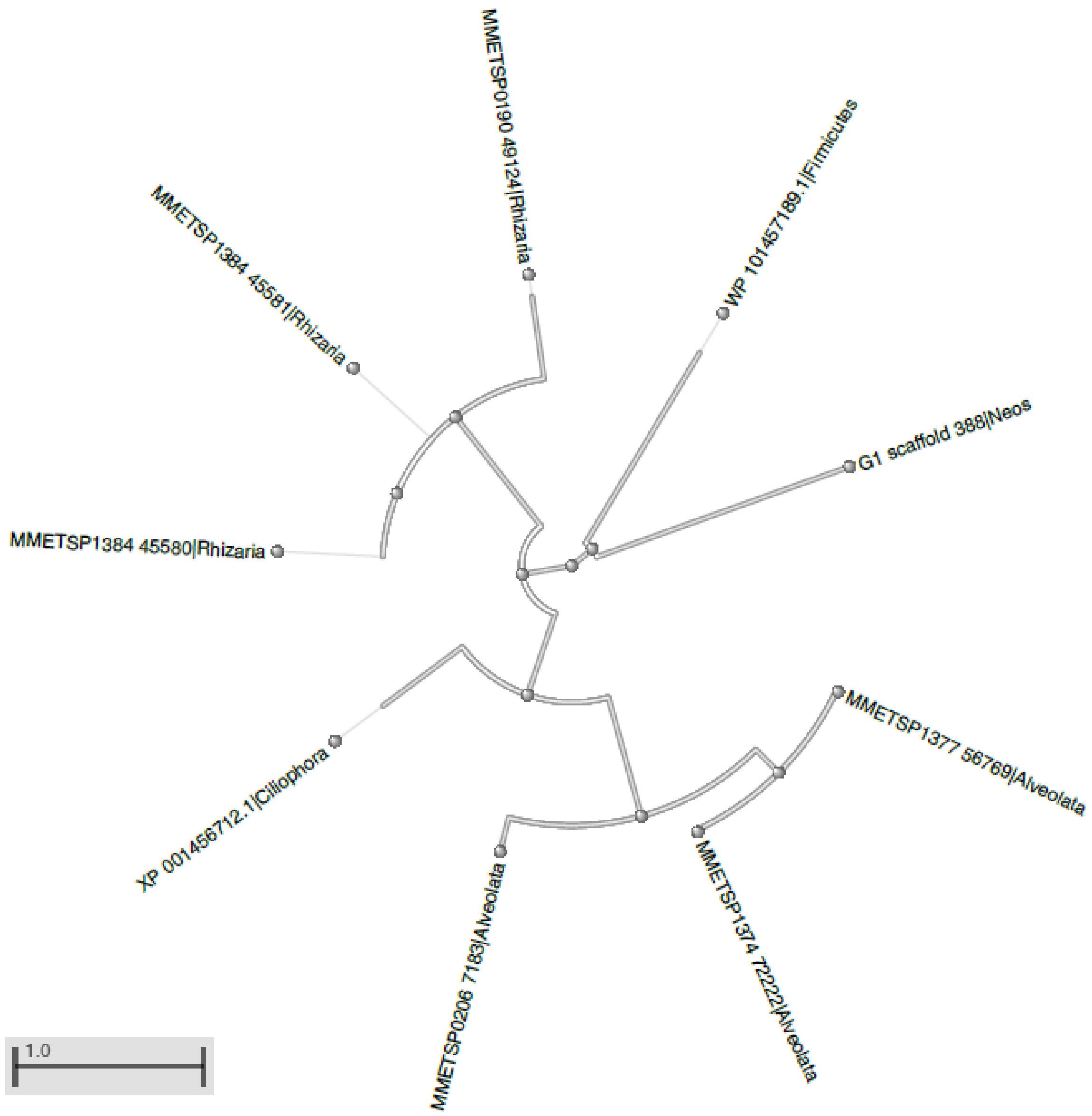

Fig. S7. Phylogeny of *N. californiae* bacteriocin located on scaffold 388 (antiSMASH cluster 5), represented as "G1 scaffold 388|Neos." Tree rendered using NCBI Tree Viewer 1.17.5.

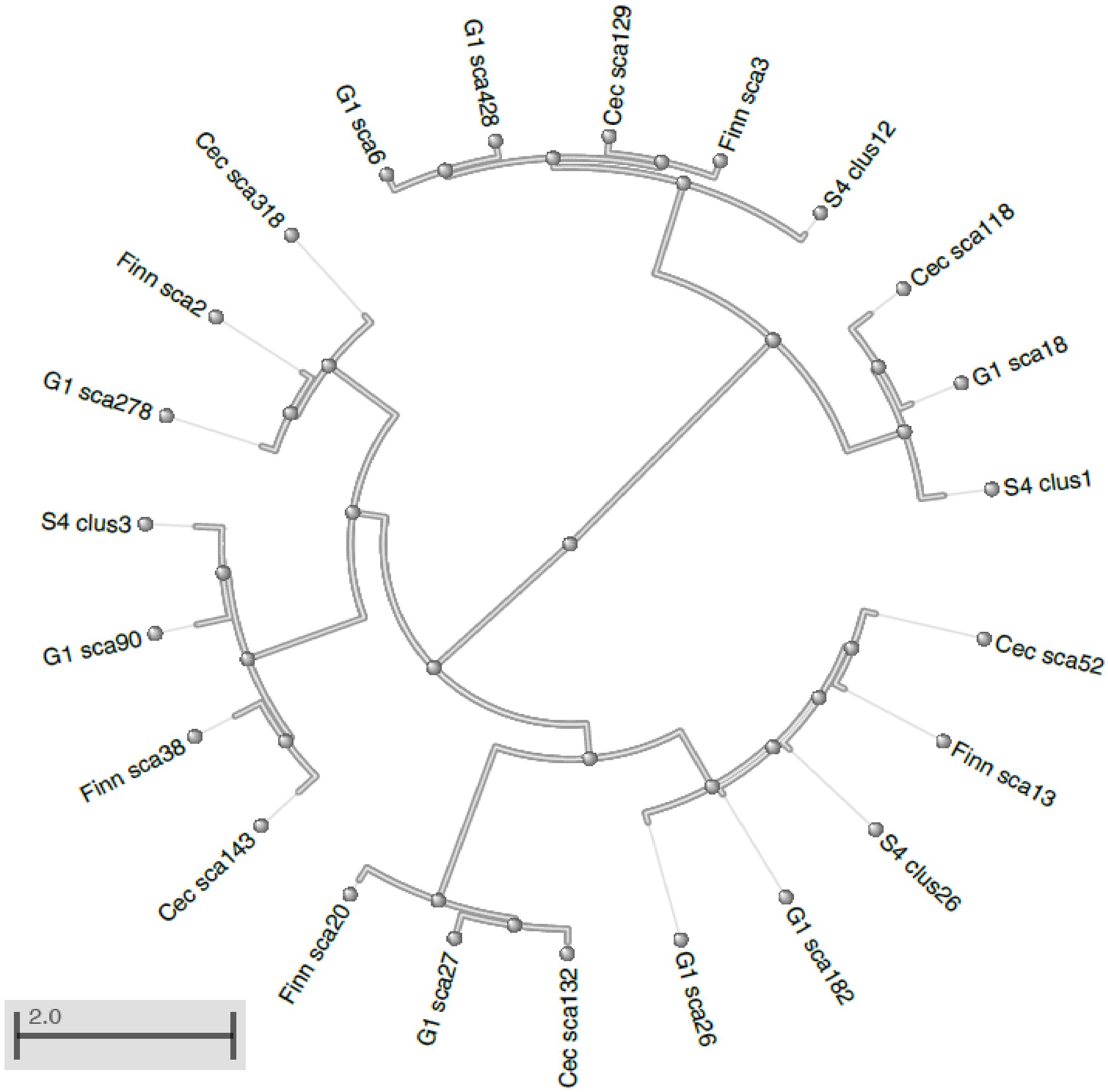

Fig. S8. Maximum likelihood phylogenetic tree of type I PKS genes from *A. robustus*, *C. churrovis*, *N. californiae*, and *P. finnis*. Each gene is represented by its scaffold or cluster number (e.g. "sca2" signifies the PKS gene on scaffold 2, "clus3" is the PKS gene in cluster 3). Cec=*C. churrovis*, G1=*N. californiae*, S4=*A. robustus*, Finn = *P. finnis*. All genes were aligned using ClustalW (11, 12). The resulting alignment file was used as input to construct a maximum likelihood phylogenetic tree by the RAxML (13) HPC2 tool on XSEDE. Default input parameters were used for both ClustalW and RAxML. Tree rendered using NCBI Tree Viewer 1.17.5.

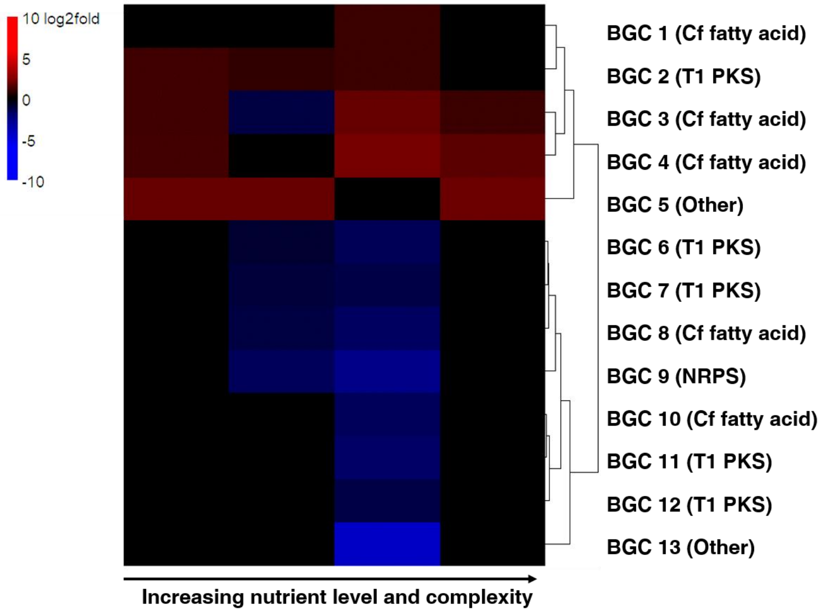

Fig. S9. Nutrient availability and complexity regulate the expression of 13 core biosynthetic genes of *N. californiae.* BGC=biosynthetic gene cluster. The heatmap shows the $\log_2$fold change of the transcript abundance of *N. californiae* grown in nutrient-poor media formulations relative to highly complex media. Only statistically significant, differentially regulated core biosynthetic genes are shown (absolute log2fold change$\geq$1, p-adjusted $\leq$ 0.01). *N. californiae* cultures were grown to early stationary phase in quadruplicate in a minimal media supplemented with nutrients of increasing level and complexity, summarized in Table S4. Columns of heatmap from left to right supplemented with: (1) no supplement ("M2"), (2) yeast extract and BactoTM Casitone ("M2 YEBC"), (3) rumen fluid ("M2 RF"), (4) yeast extract, BactoTM Casitone, and rumen fluid ("MC-").

Table S1. Comparison of members of the class Neocallimastigomycetes to other fungi from Chytridiomycota, and to representative strains of known prolific secondary metabolite producers from Ascomycota. SM=Secondary Metabolite. Each SM cluster was counted as a single gene in this estimate. Cluster counts were taken from the MycoCosm portal (3) unless otherwise noted.

Organism	SM cluster total	Gene model count	SM cluster total/gene model count [%]
**Neocallimastigomycetes**[1]			
A. robustus	46	12832	0.36
C. churrovis	16	15009	0.11
N. californiae	39	20219	0.19
P. finnis	13	10992	0.12
**Other Chytridiomycota**[2]			
B. dendrobatidis JAM81 v1.0	4	8732	0.05
R. globosum	9	16990	0.05
G. prolifera	4	13902	0.03
**Ascomycota**			
A. flavus NRRL 3357	73	12604	0.58
A. fumigatus A1163[3]	32	9916	0.32
A. nidulans[4]	72	10680	0.52
A. niger ATCC 1015 v4.0	80	11910	0.67
P. chrysogenum v1.0	50	11396	0.44

[1]Only genomes of similar quality were included for Neocallimastigomycetes.
[2]Selected members of Chytridiomycota not part of the class Neocallimastigomycetes.
[3]The estimated number of clusters for *A. fumigatus* A1163 from MycoCosm is 32, but a study of 66 *A. fumigatus* strains indicates substantially more gene clusters in *A. fumigatus* (see reference (14)).
[4]Cluster count from reference (15).

Table S2. Location of the velvet domain in putative *vosA* homologs of *C. churrovis, N. californiae,* and *P. finnis*.

Organism	MycoCosm Protein Id	Length (aa)	Velvet domain interval
C. churrovis	623244	1052	140-329
C. churrovis	624976	1090	63-243
N. californiae	112212	981	200-388
P. finnis	179530	1081	143-330

Table S3. Complete OrthoFinder (16) **results for PKS genes identified by antiSMASH** (2) **and SMURF** (17)**.** PKS genes identified by SMURF only are indicated by MycoCosm (3) protein Id, whereas genes identified by antiSMASH only or both antiSMASH and SMURF are indicated by the PKS gene scaffold location.

PKS Orthogroup	*A. robustus*	*C. churrovis*	*N. californiae*	*P. finnis*
PKS1	Scaffold_258	Scaffold_52	Scaffold_26, Scaffold_182*	Scaffold_13
PKS2	Scaffold_21	Scaffold_143	Scaffold_90	Scaffold_38
PKS3	Scaffold_5	Scaffold_118	Scaffold_18	413919
PKS4	Scaffold_127	Scaffold_129	Scaffold_6, Scaffold_428	Scaffold_3
PKS5	None	Scaffold_132	Scaffold_27	Scaffold_20
PKS6	187102	Scaffold_318	Scaffold_278	Scaffold_2

*Note that the *N. californiae* PKS gene located on scaffold 182 was grouped into a separate family by OrthoFinder. Based on the homology of neighboring genes (see additional dataset S3), it has been classified as belonging to PKS1.

Table S4. Summary of media formulations for Fig S3. Medium M2 (5) was supplemented with clarified rumen fluid, yeast extract and Bacto^{TM} Casione up to the maximum concentrations of Medium C ("MC") (18). Other media components, including trace elements, hemin, potassium phosphate, and ammonium chloride were added at equivalent amounts in all formulations.

**Component/Media**	**M2**	**M2 RF**	**M2 YEBC**	**MC-**	**MC**
Rumen fluid [vol%]	NA	7%	NA	7	1
Yeast extract [g/L]	NA	NA	0.25	0.25	2.5
Bacto^{TM} Casitone [g/L]	NA	NA	5	5	10

Table S5. Proteomics representation of the core biosynthetic genes of *P. finnis*, *A. robustus*, *C. churrovis*, and *N. californiae*. SM Class designations are the same as main text Fig. 1. T1PKS=Type 1 PKS. Gut fungal PKS families are indicated in the SM Class/Family column with the prefix "fam." Asterisk indicates that multiple core genes are present in the cluster and both were observed in the proteome. Rep=biological replicate.

Gene cluster	SM Class/Family	antiSMASH core gene	Observation counts				
			Average	Rep 1	Rep 2	Rep 3	Rep 4
P. finnis							
Finn_cluster3	Cf_saccharide	ctg4_orf11	46	47	38	54	46
Finn_cluster6	T1PKS/fam 1	ctg13_allorf0002179	44	46	36	41	53
Finn_cluster12	NRPS	ctg38_orf15	40	40	34	44	40
Finn_cluster9	Cf_fatty acid	ctg33_orf26	26	27	21	26	31
Finn_cluster14	Cf_putative	ctg59_orf9	24	22	22	24	26
Finn_cluster5	Cf_saccharide	ctg10_orf7	17	18	15	15	19
Finn_cluster8	Cf_fatty acid	ctg28_orf26	14	17	9	7	22
Finn_cluster2	T1PKS/fam 4	ctg3_orf10	10	11	8	13	7
Finn_cluster1	T1PKS/fam 6	ctg2_allorf006108	6	7	5	5	6
Finn_cluster4	Terpene	ctg5_allorf007553	3	1	4	4	3
A. robustus							
Ana_cluster37*	Cf-fatty_acid	ctg483_orf000000	58	55	73	52	50
Ana_cluster37*	Cf_fatty_acid	ctg483_orf00001	58	55	71	52	52
Ana_cluster13	Cf_fatty_acid	ctg129_orf00001	26	30	27	22	23
Ana_cluster31*	NRPS	ctg358_orf000000	17	19	16	17	16
Ana_cluster26	T1PKS/fam 1	ctg258_allorf000323	14	13	16	11	15
Ana_cluster40	NRPS	ctg540_allorf000242	9	10	9	9	6
Ana_cluster1	T1PKS/fam 3	ctg5_allorf000927	5	4	4	6	7
Ana_cluster2	Cf_saccharide	ctg13_orf003	4	4	5	4	3
Ana_cluster31*	NRPS	ctg358_allorf000041	4	4	4	4	3
Ana_cluster11	NRPS	ctg107_orf00001	3	2	3	2	3
Ana_cluster34	NRPS	ctg443_allorf000145	2	2	2	3	0
C. churrovis							
Caecom_cluster10	Cf_fatty_acid	ctg127_80	47	69	44	32	44
Caecom_cluster2	T1PKS/fam 1	ctg52_10	44	47	43	44	42
Caecom_cluster8	Cf_fatty_acid	ctg116_63	43	45	44	46	38
Caecom_cluster19	NRPS	ctg487_4	37	35	38	37	38
Caecom_cluster18	Cf_putative	ctg398_18	25	25	28	24	22
Caecom_cluster17	T1PKS/fam 6	ctg318_47	24	25	25	25	22
Caecom_cluster22	Terpene	ctg613_15	5	6	5	5	5
Caeco_cluster32	Cf_putative	ctg2951_5	3	6	4	2	1
Caecom_cluster11	T1PKS/fam 4	ctg129_32	3	1	2	3	4
N. californiae							
Neo_sca43_clus11	Cf_fatty_acid	ctg43_orf7	157	143	139	177	170
Neo_sca222_clus11	NRPS	ctg81_orf04	56	49	60	62	52
Neo_sca137_clus15	Cf_fatty_acid	ctg86_orf0002	53	48	53	47	62
Neo_sca54_clus2	Cf_saccharide	ctg3_orf5	36	36	24	45	40

Neo_sca18_clus8	T1PKS/fam 3	ctg18_orf003	25	23	25	24	27
Neo_sca239_clus12	Cf_saccharide	ctg98_orf00001	21	20	24	24	14
Neo_sca146_clus1	Cf_fatty_acid	ctg5_orf0002	14	17	16	9	15
Neo_sca172_clus5	Cf_saccharide	ctg31_orf000000	14	13	17	16	8
Neo_sca254_clus14	Cf_saccharide	ctg113_orf000000	13	13	13	16	10
Neo_sca26_clus9	T1PKS/fam 1	ctg26_allorf000372	12	15	16	12	4
Neo_sca5_clus1*	Cf_putative	ctg5_orf6	11	11	11	12	8
Neo_sca182_clus9	T1PKS/fam 1	ctg41_allorf002517	10	11	14	9	7
Neo_sca5_clus1	Cf_putative*	ctg5_orf0002	9	7	12	11	5
Neo_sca137_clus5	Cf_saccharide	ctg13_orf10	6	6	6	4	6
Neo_sca5_clus1*	Cf_putative	ctg5_orf7	5	5	9	5	1
Neo_sca6_clus2	T1PKS/fam 4	ctg6_orf20	5	6	2	7	4
Neo_sca278_clus17	T1PKS/fam 6	ctg137_allorf000524	3	3	3	2	2

Table S6. BLAST+ (9) **protein alignment of *S. baumii* PKS gene products against filtered model proteins for four species of anaerobic gut fungi.** Protein Ids refer to the MycoCosm portal (3)**.** All E-values were less than 10^{-50}.

	A. robustus			***C. churrovis***		
	Protein Id	% identity	% subject coverage	Protein Id	% identity	% subject coverage
OCB83923.1	289077	35.1	37.2	547760	35.6	37.3
OCB90292.1	328517	41.0	233.6	10344	41.2	21.5
OCB89330.1	289077	38.0	18.8	27727	34.7	22.3
OCB83944.1	289077	40.3	20.2	547760	40.5	19.0
	N. californiae			***P. finnis***		
	Protein Id	% identity	% subject coverage	Protein Id	% identity	% subject coverage
OCB83923.1	206001	34.5	30.3	413919	33.7	38.7
OCB90292.1	454950	37.4	27.9	315942	38.8	18.7
OCB89330.1	697270	33.9	21.8	413919	38.0	22.1
OCB83944.1	697270	38.8	19.3	413919	39.0	19.0

Additional dataset S1 (separate file)

Protein BLAST (version 2.2.28+) results of the bacteriocins of *C. churrovis* and *N. californiae* to the AMP database using CAMPSign (1, 19–21).

Additional dataset S2 (separate file)

BLAST results for the query of core biosynthetic genes predicted by antiSMASH 3.0 with more than three catalytic domains to the core genes of MycoCosm Secondary Metabolism clusters. Only the top bitscore hit for each query sequence is shown. Hits with percent identity greater than 93% were considered matches and are highlighted in yellow in the “pident” column.

Additional dataset S3 (separate file)

RNA-seq curated gene clusters for *A. robustus*, *C. churrovis*, *N. californiae*, and *P. finnis*. MycoCosm protein Ids are listed for each gene. Core biosynthetic genes are in bold. AntiSMASH annotations are given for the core genes and MycoCosm annotations for all others. AntiSMASH-identified PKS genes are shown for all fungi on a separate tab. Other cluster types are grouped by species. Orthologs, defined as bidirectional top-scoring BLASTp hits from filtered model proteins between genomes with E-value threshold of 10^{-5}, are given for each gene.

Additional dataset S4 (separate file)

BLAST results of the putative regulatory *C. churrovis* Protein Id 623144 containing a velvet domain queried against *N. californiae* 112212.

Additional dataset S5 (separate file)

BLAST results of the putative regulatory *C. churrovis* Protein Id 623144 containing a velvet domain queried against *P. finnis* 179530.

Additional dataset S6 (separate file)

BLAST results of the putative regulatory *N. californiae* Protein Id 112212 containing a velvet domain queried against *P. finnis* 179530.

Additional dataset S7 (separate file)

BLAST results for the query of core biosynthetic genes predicted by antiSMASH 3.0 to NCBI non-redundant databases, excluding Neocallimastigomycota. Top bitscore hits for each query highlighted in green (PKS genes) or yellow (all other genes).

Additional dataset S8 (separate file)

Predicted proteins of the biosynthetic gene clusters of *A. robustus*, *C. churrovis*, and *P. finnis* marked with dense methylated adenine clusters (MACs) within 500 bp of transcription start site.

Additional dataset S9 (separate file)

Molecular network constructed from the nonpolar metabolites extracted by ethyl acetate from the supernatant of *C. churrovis* and *P. finnis*, and control (medium C (18) incubated with reed canary grass at 39 °C). Ethyl acetate and methanol extractions were combined for the control only. Self-looping nodes are truncated. Node colors are as follows: gray=node observed in control, yellow=*C. churrovis* only, red=*P. finnis* only, orange=*C. churrovis* and *P. finnis*.

Additional dataset S10 (separate file)
Cluster from additional datasets S9 with nodes specific only to *C. churrovis* and *P. finnis*. Node colors are as follow: yellow=*C. churrovis* only, red=*P. finnis* only, orange=*C. churrovis* and *P. finnis*. Node label is mass to charge ratio.

Additional dataset S11 (separate file)
Putative natural products detected from *A. robustus* determined via Pactolus, an in-house implementation of the MIDAS (22) scoring algorithm. See Methods for further details.

Additional dataset S12 (separate file)
Putative baumin MS/MS and characteristic ions.

Additional dataset S13 (separate file)
Comparison of the peak heights of features (*m/z*, rt pairs) identified in the extracted supernatant of *A. robustus*, *C. churrovis*, *N. californiae*, or *P. finnis* to control samples (complex media containing 15% rumen fluid incubated with reed canary grass at 39 °C). Note that *A. robustus* and *N. californiae* were grown in a separate experiment from *C. churrovis* and *P. finnis* and thus have separate controls to account for rumen fluid batch variation.

Additional dataset S14 (separate file)
Protein BLAST alignment for *S. baumii* PKS OCB83923.1 against *A. robustus* PKS gene identified by antiSMASH on scaffold 127.

Additional dataset S15 (separate file)
Protein BLAST alignment for *S. baumii* PKS OCB83923.1 against the *C. churrovis* PKS gene identified by antiSMASH on scaffold 129.

Additional dataset S16 (separate file)
Protein BLAST alignment for *S. baumii* PKS OCB83923.1 against the *N. californiae* PKS gene identified by antiSMASH on scaffold 6.

Additional dataset S17 (separate file)
Protein BLAST alignment for *S. baumii* PKS OCB83923.1 against the *N. californiae* PKS gene identified by antiSMASH on scaffold 428.

Additional dataset S18 (separate file)
Protein BLAST alignment for *S. baumii* PKS OCB83923.1 against the *P. finnis* PKS gene identified by antiSMASH on scaffold 3.

References

1. G. Wang, X. Li, Z. Wang, APD3: The antimicrobial peptide database as a tool for research and education. *Nucleic Acids Res.* **44**, D1087–D1093 (2016).
2. T. Weber, *et al.*, antiSMASH 3.0--a comprehensive resource for the genome mining of biosynthetic gene clusters. *Nucleic Acids Res.*, W237–W243 (2015).
3. I. V. Grigoriev, *et al.*, MycoCosm portal: Gearing up for 1000 fungal genomes. *Nucleic Acids Res.* **42**, 699–704 (2014).
4. M. K. Theodorou, D. R. Davies, B. B. Nielsen, M. I. G. Lawrence, A. P. J. Trinci, Determination of growth of anaerobic fungi on soluble and cellulosic substrates using a pressure transducer. *Microbiology* **141**, 671–678 (1995).
5. M. J. Teunissen, H. J. M. Op den Camp, C. G. Orpin, J. H. J. Huis in 't Veld, G. D. Vogels, Comparison of growth characteristics of anaerobic fungi isolated from ruminant and non-ruminant herbivores during cultivation in a defined medium. *J. Gen. Microbiol.* **137**, 1401–1408 (1991).
6. K. V. Solomon, *et al.*, Early-branching gut fungi possess a large, comprehensive array of biomass-degrading enzymes. *Science (80-.).* **351**, 1192–1195 (2016).
7. B. Li, C. N. Dewey, RSEM: accurate transcript quantification from RNA-Seq data with or without a reference genome. *BMC Bioinformatics* **12**, 323 (2011).
8. M. I. Love, W. Huber, S. Anders, Moderated estimation of fold change and dispersion for RNA-seq data with DESeq2. *Genome Biol.* **15**, 550 (2014).
9. C. Camacho, *et al.*, BLAST plus: architecture and applications. *BMC Bioinformatics* **10**, 1 (2009).
10. M. N. Price, P. S. Dehal, A. P. Arkin, Fasttree: Computing large minimum evolution trees with profiles instead of a distance matrix. *Mol. Biol. Evol.* **26**, 1641–1650 (2009).
11. F. Sievers, *et al.*, Fast, scalable generation of high-quality protein multiple sequence alignments using Clustal Omega. *Mol. Syst. Biol.* **7**, 539 (2011).
12. F. Sievers, D. G. Higgins, Clustal Omega for making accurate alignments of many protein sequences. *Protein Sci.* **27**, 135–145 (2018).
13. A. Stamatakis, RAxML version 8: A tool for phylogenetic analysis and post-analysis of large phylogenies. *Bioinformatics* **30**, 1312–1313 (2014).
14. A. L. Lind, *et al.*, Drivers of genetic diversity in secondary metabolic gene clusters within a fungal species. *PLoS Biol.* **15**, 1–26 (2017).
15. C. G. M.T. Drott, R.W. Bastos, A. Rokas, L.N.A. Ries, T. Gabaldon, G.H. Goldman, N.P. Keller, Diversity of Secondary Metabolism in Aspergillus nidulans Clinical Isolates. *mSphere* **5**, 1–13 (2020).
16. D. M. Emms, S. Kelly, OrthoFinder: solving fundamental biases in whole genome comparisons dramatically improves orthogroup inference accuracy. *Genome Biol.* **16**, 1–14 (2015).
17. N. Khaldi, *et al.*, SMURF: Genomic mapping of fungal secondary metabolite clusters. *Fungal Genet. Biol.* **47**, 736–41 (2010).
18. M. K. Theodorou, J. Brookman, A. P. J. Trinci, "Anaerobic fungi" in *Methods in Gut Microbial Ecology for Ruminants*, (Springer-Verlag, 2005), pp. 55–66.
19. S. F. Altschul, *et al.*, Gapped BLAST and PSI-BLAST: A new generation of protein database search programs. *Nucleic Acids Res.* **25**, 3389–3402 (1997).
20. A. A. Schaffer, Improving the accuracy of PSI-BLAST protein database searches with composition-based statistics and other refinements. *Nucleic Acids Res.* **29**, 2994–3005 (2001).
21. F. H. Waghu, R. S. Barai, S. Idicula-thomas, Leveraging family-specific signatures for AMP discovery and high-throughput annotation. *Nat. Publ. Gr.*, 1–7 (2016).
22. Y. Wang, G. Kora, B. P. Bowen, C. Pan, MIDAS: A database-searching algorithm for metabolite identification in metabolomics. *Anal. Chem.* **86**, 9496–9503 (2014).
