# Supplementary figures and images for: Anaerobic gut fungi are an untapped reservoir of natural products
Source: Proc Natl Acad Sci U S A. 2021 Apr 27;118(18):e2019855118. doi: 10.1073/pnas.2019855118 (PMC8106346; doi:10.1073/pnas.2019855118)

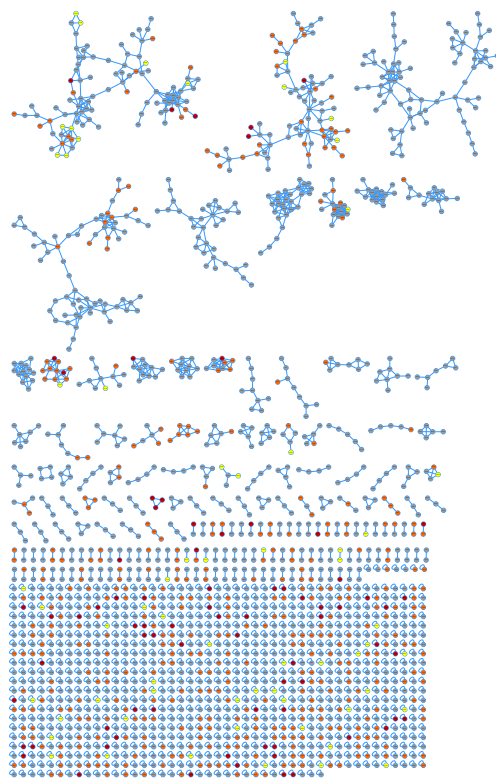

Supplement: Supplementary File [file pnas.2019855118.sd09.pdf]

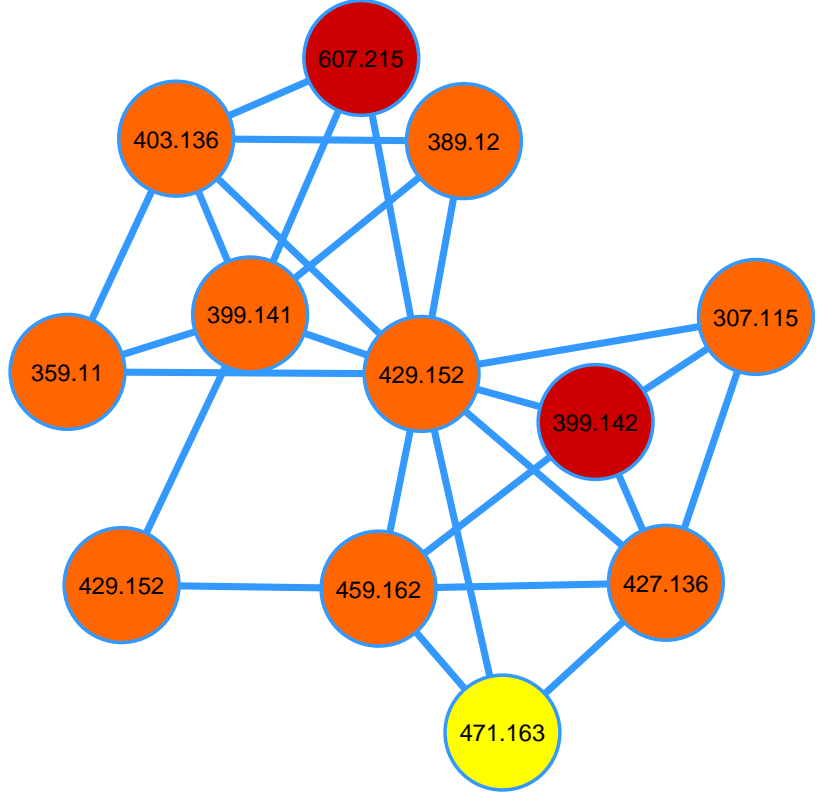

Supplement: Supplementary File [file pnas.2019855118.sd10.pdf]
