# Supplementary material for: Anaerobic gut fungi are an untapped reservoir of natural products
Source: Proc Natl Acad Sci U S A. 2021 Apr 27;118(18):e2019855118. doi: 10.1073/pnas.2019855118 (PMC8106346; doi:10.1073/pnas.2019855118)
Supplement: Supplementary File [file pnas.2019855118.sd12.pdf]

Unk_2_523p124929812_positive_4p67

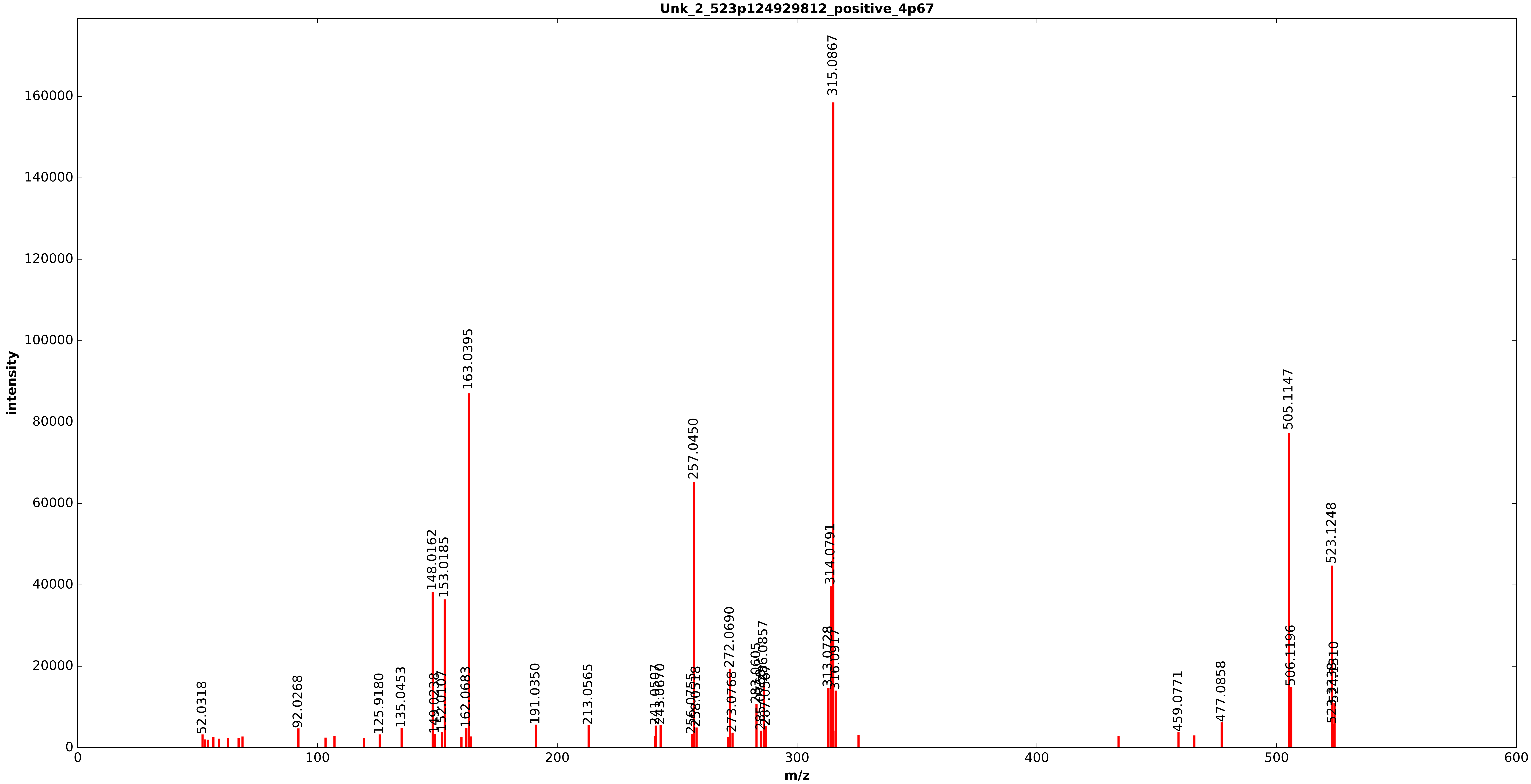

20170222_C18___POS_MSMS_KBL_MO_Qex139_UV_Fungus_1to10_17_HE_S4__ID1001B_205060eV_Run19

Unk_2_523p124929812_positive_4p67

m/z theoretical = 523.1249, measured = 523.1251, 0.2642 ppm difference

Expected Elution of 4.67 minutes, 4.67 min actual

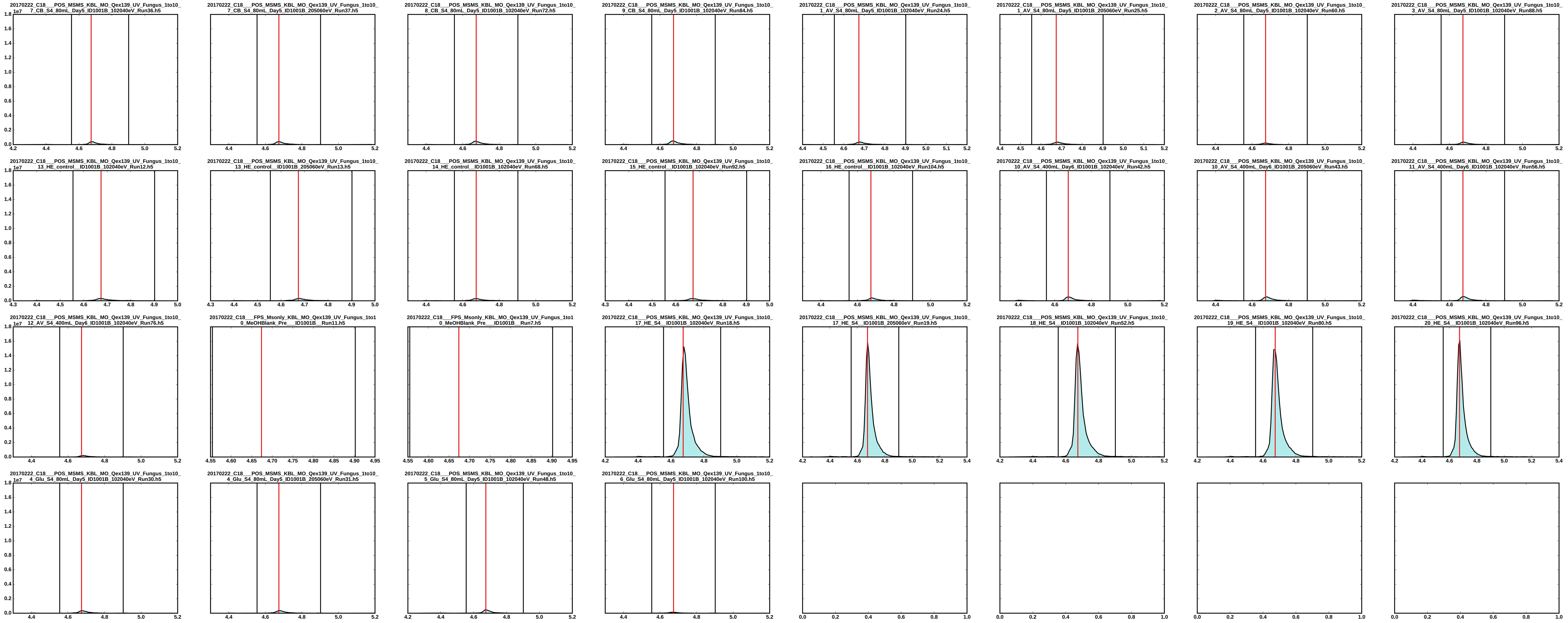
20170222_C18___POS_MSMS_KBL_MO_Qex139_UV_Fungus_1to10_ 7_CB_S4_80mL_Day5_ID1001B_102040eV_Run36.h5
20170222_C18___POS_MSMS_KBL_MO_Qex139_UV_Fungus_1to10_ 7_CB_S4_80mL_Day5_ID1001B_205060eV_Run37.h5
20170222_C18___POS_MSMS_KBL_MO_Qex139_UV_Fungus_1to10_ 8_CB_S4_80mL_Day5_ID1001B_102040eV_Run72.h5
20170222_C18___POS_MSMS_KBL_MO_Qex139_UV_Fungus_1to10_ 9_CB_S4_80mL_Day5_ID1001B_102040eV_Run84.h5
20170222_C18___POS_MSMS_KBL_MO_Qex139_UV_Fungus_1to10_ 1_AV_S4_80mL_Day5_ID1001B_102040eV_Run24.h5
20170222_C18___POS_MSMS_KBL_MO_Qex139_UV_Fungus_1to10_ 1_AV_S4_80mL_Day5_ID1001B_205060eV_Run25.h5
20170222_C18___POS_MSMS_KBL_MO_Qex139_UV_Fungus_1to10_ 2_AV_S4_80mL_Day5_ID1001B_102040eV_Run60.h5
20170222_C18___POS_MSMS_KBL_MO_Qex139_UV_Fungus_1to10_ 3_AV_S4_80mL_Day5_ID1001B_102040eV_Run88.h5
20170222_C18___POS_MSMS_KBL_MO_Qex139_UV_Fungus_1to10_ 13_HE_control__ID1001B_102040eV_Run12.h5
20170222_C18___POS_MSMS_KBL_MO_Qex139_UV_Fungus_1to10_ 13_HE_control__ID1001B_205060eV_Run13.h5
20170222_C18___POS_MSMS_KBL_MO_Qex139_UV_Fungus_1to10_ 14_HE_control__ID1001B_102040eV_Run68.h5
20170222_C18___POS_MSMS_KBL_MO_Qex139_UV_Fungus_1to10_ 15_HE_control__ID1001B_102040eV_Run92.h5
20170222_C18___POS_MSMS_KBL_MO_Qex139_UV_Fungus_1to10_ 16_HE_control__ID1001B_102040eV_Run104.h5
20170222_C18___POS_MSMS_KBL_MO_Qex139_UV_Fungus_1to10_ 10_AV_S4_400mL_Day6_ID1001B_102040eV_Run42.h5
20170222_C18___POS_MSMS_KBL_MO_Qex139_UV_Fungus_1to10_ 10_AV_S4_400mL_Day6_ID1001B_205060eV_Run43.h5
20170222_C18___POS_MSMS_KBL_MO_Qex139_UV_Fungus_1to10_ 11_AV_S4_400mL_Day6_ID1001B_102040eV_Run56.h5
20170222_C18___POS_MSMS_KBL_MO_Qex139_UV_Fungus_1to10_ 12_AV_S4_400mL_Day6_ID1001B_102040eV_Run76.h5
20170222_C18___FPS_Msonly_KBL_MO_Qex139_UV_Fungus_1to1 0_MeOHBlank_Pre___ID1001B__Run11.h5
20170222_C18___FPS_Msonly_KBL_MO_Qex139_UV_Fungus_1to1 0_MeOHBlank_Pre___ID1001B__Run7.h5
20170222_C18___POS_MSMS_KBL_MO_Qex139_UV_Fungus_1to10_ 17_HE_S4__ID1001B_102040eV_Run18.h5
20170222_C18___POS_MSMS_KBL_MO_Qex139_UV_Fungus_1to10_ 17_HE_S4__ID1001B_205060eV_Run19.h5
20170222_C18___POS_MSMS_KBL_MO_Qex139_UV_Fungus_1to10_ 18_HE_S4__ID1001B_102040eV_Run52.h5
20170222_C18___POS_MSMS_KBL_MO_Qex139_UV_Fungus_1to10_ 19_HE_S4__ID1001B_102040eV_Run80.h5
20170222_C18___POS_MSMS_KBL_MO_Qex139_UV_Fungus_1to10_ 20_HE_S4__ID1001B_102040eV_Run96.h5
20170222_C18___POS_MSMS_KBL_MO_Qex139_UV_Fungus_1to10_ 4_Glu_S4_80mL_Day5_ID1001B_102040eV_Run30.h5
20170222_C18___POS_MSMS_KBL_MO_Qex139_UV_Fungus_1to10_ 4_Glu_S4_80mL_Day5_ID1001B_205060eV_Run31.h5
20170222_C18___POS_MSMS_KBL_MO_Qex139_UV_Fungus_1to10_ 5_Glu_S4_80mL_Day5_ID1001B_102040eV_Run48.h5
20170222_C18___POS_MSMS_KBL_MO_Qex139_UV_Fungus_1to10_ 6_Glu_S4_80mL_Day5_ID1001B_102040eV_Run100.h5
1e7
